# Factors associated with water consumption among children: a systematic review

**DOI:** 10.1186/s12966-019-0827-0

**Published:** 2019-08-13

**Authors:** Carmen B. Franse, L. Wang, Florence Constant, Lisa R. Fries, Hein Raat

**Affiliations:** 1 Department of Public Health, Erasmus University Medical Center, Wytemaweg 80, 3015 CN Rotterdam, The Netherlands; 2 Nestlé Waters MT, Issy-les-Moulineaux, France; 3 Nestlé Research, Vers-chez-les-Blanc, Lausanne, Switzerland

**Keywords:** Water, Beverages, Children, Behavior, Systematic review

## Abstract

**Background:**

Water is recommended as the main beverage for daily fluid intake. Previous systematic reviews have studied the consumption of sugar-sweetened beverages (SSBs) among children, but none have focused on water consumption. Insight into factors that are associated with children’s water intake is needed to inform the development of interventions aimed at the promotion of water consumption. The objective of this review was therefore to summarize the current evidence on factors associated with water consumption among children aged 2 to 12 years.

**Methods:**

A systematic literature search in seven electronic databases was conducted in May, 2018 and retrieved 17,850 unique records. Two additional studies were identified by hand-searching references of included articles. Studies were selected if they had a cross-sectional or longitudinal study design, focused on children aged 2–12 years and published in an English language peer-reviewed journal. Participants from clinical populations, studies that included data of < 10 participants and non-human studies were excluded.

**Results:**

A total of 63 articles met inclusion criteria and were included in the analysis. We identified 76 factors that were investigated in these studies; 17/76 were investigated in a longitudinal study. There was evidence of positive associations between water consumption and child’s self-efficacy, parental education level, parental self-efficacy, use of feeding practices such as restriction or encouraging healthy eating and study year. Evidence was inconsistent (< 60% of studies reported an association) for child’s age, sex, BMI, consumption of SSBs and ethnic background of the parent. There was no evidence (≤33% of studies reported an association) of associations between consumption of milk or juice, parental emotional-, modelling- or instrumental feeding practices, eating school lunch or outside temperature and water consumption. The remaining 54 factors were investigated in fewer than three studies.

**Conclusions:**

There is some evidence for an association between potentially modifiable parental and child-related factors and water consumption. However, most factors identified in this review were only studied by one or two studies and most studies were cross-sectional. More longitudinal research is necessary to investigate environmental, parental and child-related factors associated with water consumption that are currently under-studied and could further inform intervention strategies.

**Trial registration:**

PROSPERO ID# CRD42018093362, registered May 22, 2018.

**Electronic supplementary material:**

The online version of this article (10.1186/s12966-019-0827-0) contains supplementary material, which is available to authorized users.

## Background

The rate of childhood obesity has increased dramatically in the past decades and remains a leading cause of public health concern, as overweight and obese children are at greater risk for diabetes, heart disease, and other health conditions [1–4]. In 2017, the number of overweight or obese children under the age of five was reported to be over 38 million worldwide [5]. The prevalence of overweight, including obesity, among school-aged children in the US is around 34% [6] and in European countries between 18 to 57% [7]. As childhood obesity has been shown to track into adulthood [8, 9], it is critical to develop healthy eating and drinking habits early in life.

There are many different actions that have been recommended by leading public health organizations to fight the obesity epidemic [10–12], one of which involves limiting children’s consumption of sugar sweetened beverages (SSBs). SSBs, such as soft drinks, fruit drinks and energy drinks, are currently one of the largest sources of added sugars among children [13, 14]. Greater consumption of SSBs has been associated with weight gain and obesity [15–17]. Several longitudinal studies have found that replacing SSBs with water or other non-caloric beverages slows the accumulation of body fat [18–20]. Zheng et al. who followed a cohort of 9 year old children found that daily replacement of 100 g of water for 100 g of SSBs was inversely associated with changes in BMI over 6 years [18]. Some randomized-controlled trials have been effective in both increasing water consumption and decreasing SSB consumption [21–23] or risk of overweight [24]. Adding to this, replacing SSBs with water could also reduce tooth decay as the consumption of SSBs is associated with dental caries in children and adults [25, 26]. In 2006, a guidance system for beverage consumption was developed in which water was recommended as the main beverage for daily fluid intake [27]. Since then, the American Academy of Pediatrics and the European Society for Paediatric Gastroenterology Hepatology and Nutrition have both stated that plain water should be promoted as the principal source of hydration for children and adolescents [28, 29]. However, in many countries, water makes up around half of children’s beverage intake or less; in a multi-country study across three continents, this was the case for 11/13 countries [30]. Nationally representative surveys have estimated water consumption to be 25 to 32% of total beverage intake among British children [31], 36 to 40% among US children [32], 38 to 40% among Mexican children [33], and 55 to 58% among French children [34]. In order to develop effective intervention strategies that promote water consumption among children, it is important to study which sub-populations could benefit most from these strategies and which modifiable factors these strategies could target. Currently, no overview exists on factors that are associated with water consumption among children. Previous systematic reviews have studied the factors influencing the consumption of SSBs among children [15, 16, 35], but none have focused on factors associated with water consumption.

The current review aims to identify and synthesize the evidence about the factors that influence children’s water consumption, in order to make specific recommendations about how to design interventions that could promote this behavior [35]. The socio-ecological model was applied as a framework for the factors identified in our review. The socio-ecological model describes how factors can influence a behavior from a variety of levels, including the individual level (characteristics and behavior of the child), the interpersonal level (characteristics of and interaction with parents or others), and the environmental level (characteristics of and interaction with the home, school and community), as well as the interplay between these levels [36]. At the individual level, factors that are associated with children’s food and beverage choices could be the child’s age, sex and psychological factors such as self-efficacy; in this context, this would mean the child’s confidence to be able to select healthy foods and drinks [37]. An important category of interpersonal factors are feeding practices, which are specific behaviors done by parents to influence what, when, or how much their child eats or drinks [38]; these have been shown to be associated with children’s diet [39]. The availability and accessibility of foods and beverages in the home or classroom are examples of environmental factors that could be associated with food and beverage choice in children [40–42]. The purpose of this review was therefore to summarize the current evidence on the factors associated with water consumption among children aged 2 to 12 years.

## Methods

### Search strategy

A systematic literature search was conducted in May, 2018, using the following electronic databases: Embase, Medline Ovid, Web of Science, Cochrane, PsychINFO Ovid, CINAHL EBSCOhost, and Google Scholar. A combination of the following key words were included in the search: (water or beverage* or drink* or related key words) and (child* or infant* or toddler* or related key words) and (determinant* or factor* or life-style* or diet* or parental attitude* or related key words). The search strategy was adapted to each database. The complete search strategies used are presented in Additional file 1. In addition to database searching, the references of relevant articles were screened for other potentially relevant studies. We registered the systematic review protocol for this study in the PROSPERO registry under registration number CRD42018093362 on May 22, 2018.

### Selection process

Duplicates of records retrieved in the search were removed. Title and abstract screening of the remaining records was performed by two independent researchers (CF and LW) to identify studies that met the inclusion criteria. Any disagreements at this stage were discussed between them and, if necessary, resolved by consultation with a third reviewer. Copies of full text articles were ordered for all relevant studies. Full text screening of articles was then performed by two independent researchers (CF and LW). Disagreements that arose at this stage were also resolved by consultation with a third reviewer.

### Inclusion and exclusion criteria

The criteria for including studies for this review applied in the selection process were as follows: 1) participants were children with mean age between 2 and 12 years (pre-school and primary schools age) at baseline, we did not include children aged 0–2 years because recommendations for and patterns of beverage intake change substantially over this age range (for breastmilk, water, types of milk, juice, etc.); 2) studies quantitatively assessed the association of any type of factor with water consumption, we considered factors both longitudinal determinants and cross-sectional correlates; 3) the following categories of water were included: tap water, bottled drinking water, unflavored sparkling water, flavored water (non-sugar sweetened) or any source of drinking water. Initially we included unsweetened tea without milk as a secondary outcome, however we did not find studies that measured this outcome; 4) studies had an observational design (longitudinal or cross-sectional); and 5) studies were published in an English language peer-reviewed journal, we did not limit the search to a specific time period and included all articles published since the inception of the journal. The main exclusion criteria were: 1) participants were from clinical populations (e.g. gastroenteritis, lung infections, malnutrition); 2) studies that included data of less than 10 participants; and 3) non-human studies.

### Risk of bias assessment

The risk of bias of the included studies was assessed independently by two reviewers (CF and LW) using a version of the Risk Of Bias In Nonrandomized Studies of Interventions (ROBINS-I) assessment tool that has been adapted for use in observational studies [43, 44]. As recommended by the developers of the tool, the precise definitions of the levels for the bias domains within the protocol were adapted to the current study topic and research aims, to enable homogeneity in judgement of bias (See Additional file 2). The following domains of bias were assessed: bias due to confounding, bias in the selection of participants into study, bias in classification of exposures, bias due to departures from intended exposures, bias due to missing data, bias in measurement of outcomes and bias in selection of the reported result. For each domain of bias, the study was categorized as having ‘critical’, ‘serious’, ‘moderate’, or ‘low’ risk of bias. For example, for the ‘bias due to confounding’ domain it was assessed whether confounding was to be expected in the association between the factor and water consumption and whether the study corrected for confounding variables, such as the child’s sex and age. If it was not possible to determine the risk of bias for a certain bias domain due to missing information in the article, the domain was coded as ‘no information’. More information on how each bias domain was categorized as having ‘critical’, ‘serious’, ‘moderate’, or ‘low’ risk of bias can be found in Additional file 2. The most serious rating across these bias domains determined the overall risk of bias; e.g. if a study was categorized as having a ‘moderate’ risk of bias in six domains but a ‘serious’ risk of bias in one domain, the overall risk of bias was serious. Discrepancies in the judgment of bias between the two reviewers were identified and resolved through discussion.

### Data extraction

A standardized data extraction form was developed after discussion and consensus among the study team. This standardized form was used to extract data from the included studies by a researcher (CF or LW) and all data entered in the form was checked by one of the researchers (CF). Extracted information included: year and author of study, country, study design, population and characteristics, outcome, measurement instruments used, type and level (individual, interpersonal, environmental) of factor studied, and the association between correlate/determinant and outcome. For each factor, we qualitatively described the association between correlate/determinant and water consumption (positive; negative; or no significant positive/negative association), see Additional file 3: Table S1. We considered quantitative measures of association reported in the studies such as correlation, cross-tabulation, analysis of variance and regression. When in a study analyses adjusted for confounding factors were reported, these were used. We identified three repeated cross-sectional studies and three longitudinal studies (see results section), the analyses that were used in these studies are described in Additional file 3: Table S1.

### Data synthesis

To summarize the evidence on the association of a specific factor with water consumption among children, we used a previously established method [35, 45]. The number of studies that supported the association between a specific factor and water consumption was divided by the total number of studies that examined that factor. Factors investigated by three studies or less were coded as: no association (0) when 0–33% of studies found a significant association; inconsistent association (?) when 34–59% of studies found a significant association; positive (+) or negative (−) association when 60–100% of studies found a significant association. Factors investigated by four or more studies were coded as: no association (00) when 0–33% of studies found a significant association; inconsistent association (??) when 34–59% of studies found a significant association; positive (++) or negative (−-) association when 60–100% of studies found a significant association.

## Results

### Study selection

**Fig. 1:**
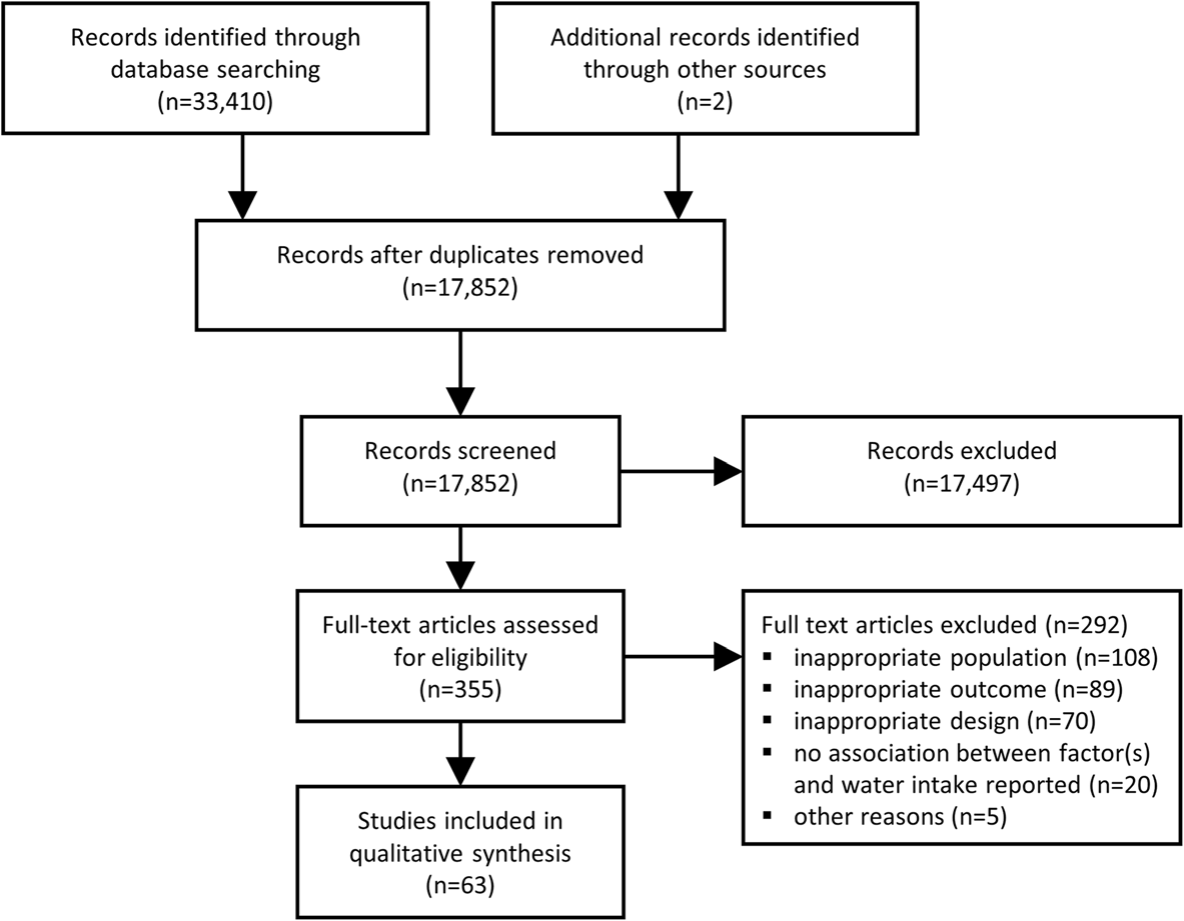
Flow chart for the selection of reviewed studies

The process of inclusion and exclusion of articles at each stage is described using the preferred reporting items of systematic reviews and meta-analyses (PRISMA) [46] flow chart (Fig. 1). A total of 33,410 records were identified after searching the seven databases. After removal of duplicates, 17,850 records remained. After all rounds of screening, 61 articles were identified. Two additional studies were identified by hand-searching the references of the included articles, resulting in a total of 63 articles that met the inclusion criteria and were included in the analysis.

### Study characteristics

The characteristics of the studies included in this review are summarized in Table 1, and details of studies can be found in Additional file 3: Table S1. From the 63 included studies, 29 studies (46%) were conducted in Europe [31, 34, 47–73] and 22 studies (35%) were conducted in North America [32, 74–94]. One study was conducted in sites in Europe, South America and Asia [30] and the remaining 11 studies were conducted in South America [33, 95–98], Australia [99–101] or Asia [102–104]. Most studies (49/63; 78%) were published in 2010 or later [30–34, 47–57, 60–64, 67–69, 71, 76, 77, 80–86, 88–90, 93, 94, 96–98, 100–103], only 2 studies (3%) were published before 2000 [65, 66]. Almost all studies (57/63; 90%) had a cross-sectional design [30–34, 47–55, 57–69, 72, 74–76, 78–99, 101–104]; 3 studies had a repeated cross-sectional design [56, 73, 77], and 3 studies had a longitudinal design [70, 71, 100].

**Table 1:** Characteristics of the studies included in the systematic review, *N* = 63

Characteristics	N of studies (%)
Place study	
Europe	29 (46)
North America	22 (35)
South America	5 (8)
Australia	3 (5)
Asia	3 (5)
Europe, South America, Asia	1 (2)
Year published	
≥ 2010	49 (78)
2000–2009	12 (19)
< 2000	2 (3)
Design	
Cross-sectional	57 (90)
Repeated cross-sectional	3 (5)
Longitudinal	3 (5)
Number of participants	
< 100	2 (3)
100–299	14 (22)
300–999	17 (27)
≥ 1000	30 (48)
Age children	
Preschool age (±2–5 years)	16 (25)
School age (±6–12 years)	25 (40)
Both age groups	22 (35)
Measure instrument water consumption	
1 day 24-h recall	20 (32)
Multi day 24-h recall	6 (10)
Food Frequency Questionnaire	18 (29)
Prospective dietary records	16 (25)
Observation researcher	3 (5)
Outcome water consumption	
Water consumption in volume/day	30 (48)
Water consumption in servings/day	21 (33)
Any water consumption (yes/no)	10 (16)
Water consumption in ml/kg body weight/day	2 (3)

The most common measure of water consumption was a single day, 24-h recall (20 studies; 32%) [33, 63, 65, 76–78, 81–84, 87, 89–92, 94, 95, 99, 100, 102], followed by Food Frequency Questionnaires (FFQ; 18 studies, 29%) [48, 50, 52, 55, 57, 61, 62, 67, 68, 71, 72, 74, 80, 85, 88, 97, 101, 103], prospective dietary records (16 studies, 25%) [30, 31, 34, 47, 49, 51, 53, 59, 60, 66, 69, 70, 73, 75, 96, 98], multiple-day 24-h recalls (6 studies, 10%) [32, 54, 64, 79, 93, 104], and behavioral observation (3 studies, 5%) [56, 58, 86].

Thirty studies (48%) reported the amount of water consumed in volume per day [30–34, 47–50, 54, 58–60, 63, 64, 67–70, 72, 73, 75, 82, 87, 88, 94–96, 98, 103], 21 studies (33%) measured water consumption in servings per day [51, 52, 55, 57, 61, 62, 66, 71, 74, 79, 80, 83–85, 89, 91, 97, 99, 101, 102, 104], 10 studies (16%) measured any water consumption (yes/no) [53, 56, 65, 77, 78, 81, 86, 90, 93, 100] and two studies measured water consumed in ml per kilo body weight per day [76, 92].

### Risk of bias

The risk of bias in each study can be found in Additional file 3: Table S2. The overall risk of bias was classified as ‘moderate’ in 8/63 studies (13%), ‘serious’ in 54/63 studies (86%) and ‘critical’ in one study (2%). The largest source of bias was due to the measurement of outcomes, with 41/63 studies (65%) being classified as having ‘serious’ risk of bias in this domain, due to reliance on one day 24 h recalls or FFQs. Almost half of the studies (29/63; 46%) were classified as having ‘serious’ risk of bias due to confounding because they did not correct for potential confounding factors, such as the child’s sex and age. Potential bias due to missing data could not be determined for 45/63 studies (71%), due to the studies not reporting how much data was missing and/or differences between included and excluded participants. Risk of bias in the selection of participants into the study, in the classification of exposures and in the selection of the reported result was relatively low compared to the other bias domains (73, 88 and 84% of studies were classified as having a ‘low’ or ‘moderate’ risk of bias in these categories, respectively).

### Factors associated with water consumption in children

Table 2 provides an overview of all factors associated with water consumption in children that were investigated in the 63 studies. Details of the associations can be found in Additional file 3: Table S1. Of the 76 factors identified, 55 (72%) of the factors were investigated by one or two studies, 10 (13%) of the factors were studied by 3 studies and 11 (14%) of the factors were studied by 4 or more studies. Among the total of 76 factors, only 17 factors (22%) were studied in a longitudinal study. Results are presented in the context of the socio-ecological framework, using the following categories: individual factors, interpersonal factors, and environmental factors.

**Table 2:** Evidence of 63 included studies on the association between factors and water consumption among children

Factor	Negative association	No association	Positive association	n/N^a^	Summary^b^
Individual level					
Socio-demographic					
Age	Beltrán-Aguilar; Sohn	**Cockburn**; Coppinger; Fenandez-Alvira, 2014; Patel, 2014; Petter; Vieux, 2017; Wang	Barraj; Drewnowski; Feferbaum; Jomaa; Patel, 2013; Piernas; Vieux, 2016	7/16	??
Sex (girl)	Jomaa; Lioret; Papandreou; Patel, 2014; Pinket 2016b; Piernas(4-8y)^c^; Vieux, 2016	Beltrán-Aguilar; Bougatsas; Campos; Coppinger; Drewnowski; Fenandez-Alvira, 2014; Piernas(9–13y)^c^; Sichieri; Sohn; Vieux, 2017; **Zohouri**	**Cockburn**	8/19	??
Health					
BMI		Dodd; Jomaa; Maffeis; Sichieri; Vieux, 2017	Cardon; Papandreou; **Sleddens**	3/8	??
Medical condition		**Cockburn**		0/1	0
Psychosocial					
Knowledge			Murnan	1/1	+
Expectations of drinking water			Sharma	1/1	+
Desire to drink any beverage		Lora		0/1	0
Intention to drink water			Patel, 2014	1/1	+
Preference water			Cullen	1/1	+
Preference sugar-sweetened beverages		Cullen		0/1	0
Self-efficacy drinking water			Dai; Elmore; Murnan	3/3	+
Self-control drinking water			Elmore	1/1	+
Behavior					
Sleep duration		Franckle		0/1	0
Physical activity		Jomaa	Senterre	1/2	?
Consumption behavior					
Consumption fruit/vegetables			Terry	1/1	+
Consumption milk		Danyliw; Terry	Sichieri	1/3	0
Consumption sugar-sweetened beverages	Mantziki 2017; Terry	Danyliw; Sichieri		2/4	??
Consumption juice		Danyliw; Mantziki 2017; Sichieri; Terry		0/4	00
Consumption moisture in drinks	Kant			1/1	–
Consumption energy		Kant		0/1	0
Consumption amount	Kant(2-5y)^c^		Kant(6-11y)^c^	1/2	?
Consumption fat		Kant		0/1	0
Consumption protein		Kant		0/1	0
Consumption carbohydrate		Kant		0/1	0
Consumption sugars	Kant			1/1	–
Consumption fiber		Kant(2-5y)^c^	Kant(6-11y)^c^	1/2	?
Consumption sodium		Kant		0/1	0
Number of eating occasions		Kant	Kakietek	1/2	?
Consumption snack		Kant(2-5y)^c^; Terry	Kant(6-11y)^c^	1/3	0
Having breakfast		Kant(2-5y)^c^	Kant(6-11y)^c^	1/2	?
Interpersonal level					
Parental socio-demographic					
Education level (lower)	Ebenegger; Fernández-Alvira, 2013; Pinket 2016b	Mantziki, 2015; Jomaa		3/5	–
Income (lower)	Vieux, 2017	Beltrán-Aguilar; Drewnowski; Jomaa; Vieux, 2016		1/6	00
Socioeconomic status indicator^d^ (lower)	**Cockburn**; Terry	Campos; Cunningham; Jomaa; Makkes; Milla Tobarra; Patel, 2014	Sohn	2/9	00
Ethnic background/race (non-white)	**Cockburn**; Drewnowski; Patel, 2014	Beltrán-Aguilar; Dodd; Ebenegger; Vieux, 2017	Sohn	3/8	??
Generation immigration (first)			Parsons	1/1	+
Language (not English)		**Cockburn**	Patel, 2014	1/2	?
Receives nutritional support		Watowicz		0/1	0
Parental psychosocial					
Knowledge		Pinket,2016a		0/1	0
Self-efficacy			Campbell; Mantziki, 2017; Pinket,2016a	3/3	+
Perceives barriers	Cullen	Lora		1/2	?
Concern weight child		Lora		0/1	0
Parent-child interaction					
Communicating health belief			Mantziki 2017	1/1	+
Controlling feeding practice		Inhulsen; **Sleddens**		0/2	0
Emotional feeding practice		Inhulsen; Lora; Mantziki, 2017; **Sleddens**;	Pinket,2016a	1/5	00
Restrictive feeding practice			Mantziki 2017; Pinket,2016a; **Sleddens**	3/3	+
Modelling feeding practice		Mantziki 2017; Pinket,2016a	**Sleddens**	1/3	0
Negotiating feeding practice			Mantziki 2017	1/1	+
Encouraging feeding practice		**Sleddens**	Inhulsen; Pinket,2016a	2/3	+
Instrumental feeding practice	Inhulsen	Lora; **Sleddens**		1/3	0
Pressure feeding practice		**Sleddens**		0/1	0
Monitoring feeding practice		Mantziki 2017; **Sleddens**		0/2	0
Environmental level					
Home					
Availability soft drinks	Mantziki 2017; Pinket,2016a			2/2	–
Availability fruit juice	Pinket,2016a	Mantziki 2017		1/2	?
Availability water			Pinket,2016a	1/1	+
School					
Free access water in classroom			Kaushik	1/1	+
Having school lunch	Condon	Dubuisson	Evans	1/3	0
School overall		Vereecken		0/1	0
School compliant water regulations		Kakietek		0/1	0
School participates nutritious meals	Kaketiek			1/1	–
School participates nutrition training		Kakietek		0/1	0
School participates program targeted low income families			Kaketiek	1/1	+
School operating hours			Kakietek	1/1	+
Classroom size		Kakietek		0/1	0
Student-teacher ratio		Kakietek		0/1	0
Teaching staff turnover			Kakietek	1/1	+
Consumption place/time					
Eating at other’s house		Ayala		0/1	0
Eating at restaurant	Ayala			1/1	–
Type of restaurant		Ayala		0/1	0
Meal time (lunch)			Campos	1/1	+
Consumption during meal			Fenandez-Alvira, 2014	1/1	+
Consumption on weekday			Hoffmann	1/1	+
Other					
Country			De Craemer; Guelinckx	2/2	+
Region		**Cockburn**	Vieux, 2017	1/2	?
Outside temperature		Sohn; Terry	Beltrán-Aguilar	1/3	0
Season (summer)		Vieux, 2017	Barraj	1/2	?
Time			**Bleich; Haroun; Sichert-Hellert; Zohouri**	4/4	++

Longitudinal and repeated cross-sectional studies are shown in bold. a) n = number studies reporting significant association; N = total number studies investigating association. b) For 3 studies: (0) no association, 0–33% of studies showed a significant association; (?) inconsistent association, 34–59% of studies reported significant associations; (+) positive or (−) negative association, 60–100% of studies demonstrated significant associations. For 4 or more studies a summary of these associations is presented with (00), (??), (++), or (−-) respectively. c) These studies stratified associations between factor and water consumption by age group, when associations were different, results are presented by age group and counted as 2 studies. d) Public/private school (2 studies), socio-economic index for areas, food insecurity, eligibility free/reduced lunch, health care card recipients, poverty-income ratio, employment status, index based on education and occupation

### Individual factors

Thirty individual level factors were identified, of which 22 factors were only studied in one or two studies. Four factors were studied in a longitudinal study. There was evidence for a positive association between the child’s self-efficacy in consuming enough water and water consumption (3/3 studies; all cross-sectional). One cross-sectional study found a positive association between consumption of fruit or vegetables and water consumption and one cross-sectional study found a negative association between consumption of sugar and water consumption. There was inconsistent evidence for positive associations between the child’s age and water consumption (7/16 studies; 15 cross-sectional 1 longitudinal) and between the child’s body mass index (BMI) and water consumption (3/8 studies; 7 cross-sectional 1 longitudinal). There was also inconsistent evidence for girls consuming less water (8/19 studies; 18 (repeated) cross-sectional 1 longitudinal). There was inconsistent evidence for a negative association between consumption of SSBs and water consumption (2/4 studies; all cross-sectional) and no evidence of an association between milk consumption (1/3 studies; all cross-sectional) or juice consumption (0/4 studies; all cross-sectional) and water consumption.

### Interpersonal factors

Twenty-one interpersonal level factors were identified, of which 11 factors were only studied in one or two studies. In total, 11 factors were studied in a longitudinal study. There was evidence for a positive association between parent’s education level and the child’s water consumption (3/5 studies; all cross-sectional). In contrast, there was no evidence of an association between family income (1/6 studies; all cross-sectional) or other indicators of socioeconomic status (2/9 studies; 8 cross-sectional 1 longitudinal) and child’s water consumption. There was evidence for a positive association between self-efficacy of the parents regarding healthy nutrition and child’s water consumption (3/3 studies; all cross-sectional). Among the parental feeding practices, there was evidence for positive associations between restriction (3/3 studies; 2 cross-sectional 1 longitudinal) and encouraging healthy eating/drinking (2/3 studies; 2 cross-sectional 1 longitudinal) and the child’s water consumption. There was inconsistent evidence that children of parents with a non-white background consume less water (3/8 studies; 7 cross-sectional 1 longitudinal). There was no evidence for emotional feeding practices (1/5 studies; 4 cross-sectional 1 longitudinal), modelling (1/3 studies; 2 cross-sectional 1 longitudinal), instrumental feeding practices (1/3 studies; 2 cross-sectional 1 longitudinal).

### Environmental factors

Twenty-five environmental level factors were identified, of which 22 factors were only studied in one or two studies. Two factors were studied in a longitudinal study. There was evidence for an increasing trend in children’s water consumption in more recent study years compared to earlier study years (4/4 studies; 3 repeated cross-sectional 1 longitudinal). There was some evidence for country differences in water consumption among children (2/2 studies; both cross-sectional). There was some evidence for a negative association between home availability of soft drinks and water consumption (2/2 studies; both cross-sectional). Two cross-sectional studies found positive associations between the availability of water and water consumption: one focusing on availability in the home, and the other on free access to water in the classroom. Evidence for most factors relating to school nutrition policies was inconsistent and studied by single studies. There was no evidence for an association between having school lunch and water consumption (1/3 studies; all cross-sectional).

## Discussion

This review aimed to summarize the evidence of factors associated with water consumption among children aged 2–12 years. A large number of factors at the individual, interpersonal and environmental levels were identified and there was evidence that several factors were associated with water consumption in children. However, the majority of factors were only investigated by one or two studies and most studies were cross-sectional. Research on childhood water consumption appears to be a relatively new field as more than three-quarters of the studies identified were published in 2010 or later. Many older studies on beverage consumption did not measure water consumption [105]. However, several interventions have aimed to replace children’s consumption of SSBs by water [20, 21, 106]. This highlights the importance of studying the factors associated with water consumption in children, alongside the factors associated with SSB consumption, as the drivers, motivators, and barriers may differ between beverage categories.

### Individual factors

There was consistent evidence for a positive association between both the child’s self-efficacy to drink enough water and water consumption. Self-efficacy has also been associated with other healthy dietary behaviors and prevention of weight gain [41, 107]. Although, to our knowledge, there have not been any interventions targeting self-efficacy in order to promote water intake, this could be a promising approach. In the domain of nutrition, a Canadian intervention that included peer-based healthy living lessons among primary-school children found a significant increase in self-efficacy, and also an improvement in dietary intake [108].

The evidence for an association between the child’s age and sex and water consumption was inconsistent. This could partly be due to differences in measurement of water intake. The seven studies that found a positive association between age and water consumption generally measured water consumption in volume per day, whereas the two studies that found a negative association between the child’s age and water consumption measured water consumption in volume per kilogram of bodyweight per day. In addition, around half of all studies included in the review measured children’s water consumption in number of servings per day or as a bivariate outcome (consumed water or not). As water intake recommendations are expressed in liters or milliliters per day [109, 110], it would be valuable for future studies to use these measures in order to increase comparability between studies, and to dietary guidelines.

The evidence for a negative association between SSB consumption and water consumption was mixed and there was no evidence of associations between consumption of milk or juice and water consumption in children. More research needs to be done on the interrelation between the consumption of different types of beverage categories such as SSBs (e.g. soft drinks, fruit drinks and energy drinks), juice and milk among children. It is unclear if and when water consumption replaces the consumption of other beverages or whether water is consumed in addition to other beverages in non-experimental settings.

We found mixed evidence for a positive association between BMI and water consumption. Children with a higher BMI may consume both more water as well as SSBs compared with children with a lower BMI, which is found in some studies [31, 111]. However, other studies have found non-significant differences in overall beverage consumption patterns according to weight status [60, 81]. Interestingly, diet drink consumption has sometimes also been found to be higher among overweight persons [81, 112]. It may be possible that overweight children compensate calorie intake from solid foods by drinking water.

### Interpersonal factors

Restrictive parenting practices towards unhealthy nutrition and encouraging parenting practices towards healthy nutrition were associated with higher water consumption in children. Of the three studies that measured the association between parental modelling and the child’s water consumption, only the one longitudinal study found an association. The broader literature generally identifies parent’s restrictive-, encouraging-, and modelling practices as beneficial to children’s diet quality, although findings are mixed [35, 41, 113]. However, different feeding practices may be required to promote intake of healthy foods and drinks than those that decrease intake of unhealthy foods, thus findings related to water intake may more closely reflect those related to healthy food intake (e.g., fruits and vegetables), rather than those related to unhealthy beverages (e.g., SSBs). Further, different feeding practices may be appropriate for younger versus older children, thus potentially contributing to some mixed findings in the literature [113]. Promoting specific parental feeding practices appears to be a promising strategy for the promotion of water consumption among children, although more studies need to be done to determine the specific feeding practices that are the most beneficial.

Similar to our findings for children’s self-efficacy, there was also consistent evidence for a positive association between the parent’s self-efficacy towards healthy nutrition and the child’s water consumption. A Dutch parenting intervention among parents of overweight and obese children found that parent’s self-efficacy was modifiable, and found positive effects on children’s soft drink consumption [114]. It remains to be explored how parent’s self-efficacy can be addressed with respect to encouraging children to consume water more often.

With regard to demographic factors, we found evidence for an association between parental education level and child’s water consumption, but no evidence for family income or other indicators of socioeconomic status. The findings related to ethnic background were inconclusive. Other reviews also found mixed evidence regarding the association between socioeconomic status or ethnic background and healthy food and energy-balance behaviors [35, 115].

### Environmental factors

Environmental factors relating to water consumption in children appear to be largely understudied. The most consistent evidence was found for an increasing trend in children’s water consumption over time. The most recent of these studies was done in the US and found an increase in children’s water consumption from 2004 to 2014; as well as a decreasing trend in children’s SSB consumption [77]. Among public health efforts that could have impacted on this trend, the authors mention beverage taxes that were implemented in several states across the US [77].

Some studies included in our review found that availability and access to water at home or at school was associated with higher water consumption- and availability of SSBs with lower water consumption. Availability and accessibility have also been consistently associated with fruit and vegetable consumption in children [40–42]. Giving children free access to water during school hours could be a key strategy to promote children’s water consumption. The association between school nutrition policies and water consumption in children was only studied by single studies. The relationship between school nutrition policies and children’s water consumption could be a promising field for further study.

### Strengths and limitations

To our knowledge, this was the first systematic review to investigate factors associated with water consumption in children. Previous reviews have focused on factors associated with SSB consumption in children and intervention studies aiming to reduce SSB intake [35, 116]. We performed an extended literature search in seven databases and followed a rigorous procedure for the selection of studies [117]. In addition, the references of included studies were hand-searched, which resulted in the inclusion of two additional studies. Some limitations of our review must also be acknowledged. Because we only included published studies, there is a possibility of publication bias in the findings of this review [118]. Furthermore, we only studied articles published in English and thus might have missed studies that were published in other languages. Also, there were not enough studies done on each factor to be able to stratify our results by age group. However, factors associated with water consumption might vary according to children’s age. Most studies included in this review had a cross-sectional study design, therefore reverse causation cannot be excluded. For example, while a parental feeding practice could impact the child’s eating and drinking behavior, the child’s eating habits could also influence the feeding practices parents adopt [119, 120]. We found indications for potential bias in most of the studies. This was largely due to potential bias in the applied measurements of water consumption, where many studies relied on retrospective self-reported dietary data. Furthermore, studies among younger children relied on parental report of children’s consumption of water. These methods have been found to be imprecise due to underreporting of food and beverage intake because of poor recall of the actual amounts consumed [121, 122]. Quantities of water, in particular, may be underreported as it is often consumed outside of regular mealtimes and over the course of the day. These measures may also be biased due to children and their parents giving socially desirable answers [121, 123]; that is to say, (parents of) children with a low water consumption could be tempted to over report the water consumption.

## Conclusions

A large number of factors at the individual, interpersonal and environmental level were identified that were associated with water consumption, however many of these factors were studied by only one or two studies. There is some evidence for an association between potentially modifiable factors (parental and child self-efficacy and specific parental feeding practices) and water consumption, however most of this evidence comes from cross-sectional studies. More research is necessary to investigate environmental, parental and child-related factors that are currently under-studied and could further inform intervention strategies.

## Additional files

Additional file 1: Search strategy of the review on factors associated with water consumption among children. (DOCX 18 kb)

Additional file 2: ROBINS-I risk of bias protocol specified for the review on factors associated with water consumption among children. (DOCX 25 kb)

Additional file 3: Characteristics, associations and risk of bias of studies included in the review on factors associated with water consumption among children. (DOCX 85 kb)

## Data Availability

Not applicable.

## Abbreviations

BMI: Body mass index
PRISMA: Preferred reporting items of systematic reviews and meta-analyses
ROBINS-I: Risk Of Bias In Nonrandomized Studies of Interventions
SSB: Sugar sweetened beverage
