# Supplementary material for: Factors associated with water consumption among children: a systematic review
Source: Int J Behav Nutr Phys Act. 2019 Aug 13;16:64. doi: 10.1186/s12966-019-0827-0 (PMC6693220; doi:10.1186/s12966-019-0827-0)
Supplement: Supplementary file 1 — Search strategy of the review on factors associated with water consumption among children. (DOCX 18 kb) [file 12966_2019_827_MOESM1_ESM.docx]

| **Databases** | **Before de-duplication** | **After de-duplication** |
| --- | --- | --- |
| embase.com | 9249 | 9078 |
| Medline Ovid | 8760 | 3565 |
| PsycINFO Ovid | 1443 | 314 |
| CINAHL EBSCOhost | 4286 | 1161 |
| Cochrane CENTRAL | 685 | 114 |
| Web of science | 8787 | 3502 |
| Google scholar | 200 | 116 |
| **Total** | **33410** | **17850** |

**embase.com 9249**

('fluid intake'/exp OR 'beverage'/de OR 'carbonated beverage'/de OR 'carbonated water'/de OR 'drinking water'/de OR 'mineral water'/de OR 'soft drink'/de OR 'sports drink'/de OR 'sweetened beverage'/exp OR 'fruit juice'/exp OR 'fruit and vegetable juice'/de OR tea/de OR 'energy drink'/de OR (((water OR tapwater OR fluid* OR beverage* OR liquid*) NEXT/1 (drinking)) OR ((water OR fluid* OR beverage* OR liquid* OR juice OR drink* OR tea) NEAR/6 (intake* OR consum*)) OR ((carbonat* OR fizzy OR soda OR seltzer OR co2 OR co-2 OR bubble OR sugar* OR nonsugar* OR sweet* OR nonalcohol* OR non-alcohol* OR energy OR soft OR fruit OR sucralose* OR neotame* OR acesulfame* OR saccharin* OR sport*) NEAR/3 (beverage* OR water OR drink*)) OR 'fruit juice*' OR soda OR lemonade OR (bottle* NEAR/3 water)):ab,ti) AND ('child'/de OR 'preschool child'/de OR 'school child'/de or 'primary school'/de OR 'child behavior'/de OR 'child health'/de OR 'child nutrition'/de OR childhood/de OR 'childhood obesity'/de OR (child* OR toddler* OR pre-school* OR preschool* OR schoolchild* OR primary-school* OR elementary-school* OR kindergar*):ab,ti) AND ('risk factor'/exp OR 'social aspects and related phenomena'/exp OR 'social determinants of health'/exp OR 'parent'/exp OR 'lifestyle'/exp OR 'behavior change'/exp OR 'environmental factor'/exp OR 'parental behavior'/exp OR 'child parent relation'/exp OR 'health care policy'/de OR government/de OR 'family life'/de OR advertising/de OR 'television viewing'/de OR 'eating habit'/de OR 'feeding behavior'/de OR 'parental attitude'/exp OR demography/exp OR tax/de OR prevention/exp OR 'health promotion'/exp OR 'health education'/exp OR 'community program'/exp OR 'mass medium'/exp OR 'review'/exp OR 'intervention study'/de OR 'evaluation study'/exp OR 'clinical trial'/exp OR 'caregiver'/de OR (determinant* OR influen* OR factor* OR social OR socio* OR parent* OR mother* OR father* OR maternal* OR paternal* OR lifestyle* OR life-style* OR ((behav* OR habit* OR pattern* OR practic*) NEAR/3 (change* OR diet* OR feeding OR food OR snack* OR intervent* OR health* OR unhealth* OR eating)) OR (environment* NEAR/3 (factor* OR condition* OR home OR school)) OR adverti* OR policy OR policies OR government* OR income OR poverty OR neighbourhood OR neighborhood OR communit* OR sedentar* OR tax OR taxes OR television OR (screen NEAR/3 (view* OR time)) OR Prevent* OR reduc* OR increas* OR promot* OR education OR curriculum OR program* OR polic* OR media OR television OR campaign* OR review* OR intervention* OR initiative* OR strateg* OR evaluation OR trial* OR effect* OR impact* OR ((parent OR role) NEAR/3 model*) OR caregiver* OR ((access OR availab* OR quality OR supply) NEAR/3 (water OR drinkwater))):ab,ti) NOT ([animals]/lim NOT [humans]/lim) NOT ('alcohol consumption'/exp OR ((alcohol OR binge) NEAR/3 (drinking OR consum*)):ab,ti) NOT ([Conference Abstract]/lim OR [Letter]/lim OR [Note]/lim OR [Editorial]/lim) AND [english]/lim

**Medline Ovid 8760**

(Beverages/ OR exp Carbonated Beverages/ OR exp Drinking Water/ OR exp Drinking/ OR exp Mineral Waters/ OR Fruit and Vegetable Juices/ OR Tea/ OR exp Energy Drinks/ OR (((water OR tapwater OR fluid* OR beverage* OR liquid*) ADJ (drinking)) OR ((water OR fluid* OR beverage* OR liquid* OR juice OR drink* OR tea) ADJ6 (intake* OR consum*)) OR ((carbonat* OR fizzy OR soda OR seltzer OR co2 OR co-2 OR bubble OR sugar* OR nonsugar* OR sweet* OR nonalcohol* OR non-alcohol* OR energy OR soft OR fruit OR sucralose* OR neotame* OR acesulfame* OR saccharin* OR sport*) ADJ3 (beverage* OR water OR drink*)) OR fruit juice* OR soda OR lemonade OR (bottle* ADJ3 water)).ab,ti.) AND (exp Child/ OR exp Infant/ OR exp Adolescent/ OR exp "Child Behavior"/ OR exp "Parent Child Relations"/ OR exp "Pediatrics"/ OR "Child Nutrition Sciences"/ OR "Infant nutritional physiological phenomena"/ OR "Child Rearing"/ OR "Child Psychology"/ OR (child* OR toddler* OR pre-school* OR preschool* OR schoolchild* OR primary-school* OR kindergar*).ab,ti.) AND (exp Risk Factors/ OR exp Sociological Factors/ OR exp Social Determinants of Health/ OR exp Parents/ OR exp Life Style/ OR exp Parent-Child Relations/ OR exp Health Policy/ OR exp Government/ OR exp Family Relations/ OR Advertising as Topic/ OR Television/ OR exp Feeding Behavior/ OR exp Demography/ OR Taxes/ OR exp preventive medicine/ OR exp Health Promotion/ OR exp Health Education/ OR Review/ OR Review Literature as Topic/ OR Evaluation Studies/ OR Evaluation Studies as Topic/ OR exp Clinical Trial/ OR Clinical Trials as Topic/ OR Caregivers/ OR (determinant* OR influen* OR factor* OR social OR socio* OR parent* OR mother* OR father* OR maternal* OR paternal* OR lifestyle* OR life-style* OR ((behav* OR habit* OR pattern* OR practic*) ADJ3 (change* OR diet* OR feeding OR food OR snack* OR intervent* OR health* OR unhealth* OR eating)) OR (environment* ADJ3 (factor* OR condition* OR home OR school)) OR adverti* OR policy OR policies OR government* OR income OR poverty OR neighbourhood OR neighborhood OR communit* OR sedentar* OR tax OR taxes OR television OR (screen ADJ3 (view* OR time)) OR Prevent* OR reduc* OR increas* OR promot* OR education OR curriculum OR program* OR polic* OR media OR television OR campaign* OR review* OR intervention* OR initiative* OR strateg* OR evaluation OR trial* OR effect* OR impact* OR ((parent OR role) ADJ3 model*) OR caregiver* OR ((access OR availab* OR quality OR supply) ADJ3 (water OR drinkwater))).ab,ti.) NOT (exp animals/ NOT humans/) NOT (alcohol consumption/ OR ((alcohol OR binge) ADJ3 (drinking OR consum*)).ab,ti.) NOT (letter* OR news OR comment* OR editorial* OR congres* OR abstract* OR book* OR chapter* OR dissertation abstract*).pt. AND english.la.

**PsycINFO Ovid 1443**

(fluid intake/ OR "Beverages (Nonalcoholic)"/ OR exp Water Intake/ OR (((water OR tapwater OR fluid* OR beverage* OR liquid*) ADJ (drinking)) OR ((water OR fluid* OR beverage* OR liquid* OR juice OR drink* OR tea) ADJ6 (intake* OR consum*)) OR ((carbonat* OR fizzy OR soda OR seltzer OR co2 OR co-2 OR bubble OR sugar* OR nonsugar* OR sweet* OR nonalcohol* OR non-alcohol* OR energy OR soft OR fruit OR sucralose* OR neotame* OR acesulfame* OR saccharin* OR sport*) ADJ3 (beverage* OR water OR drink*)) OR fruit juice* OR soda OR lemonade OR (bottle* ADJ3 water)).ab,ti.) AND (100.ag. OR 200.ag. OR "Child Psychology"/ OR (child* OR toddler* OR pre-school* OR preschool* OR schoolchild* OR primary-school* OR kindergar*).ab,ti.) AND (exp Risk Factors/ OR exp Sociocultural Factors/ OR exp Socioeconomic Status/ OR exp Parents/ OR exp LifeStyle/ OR exp Parent Child Relations/ OR exp Health Care Policy/ OR exp Government/ OR exp Family Relations/ OR Advertising/ OR Television/ OR Television viewing/ OR Mass media/ OR exp Food intake/ OR exp Eating behavior/ OR exp Demographic Characteristics/ OR Taxation/ OR exp preventive medicine/ OR prevention/ OR exp Health Promotion/ OR exp Health Education/ OR "literature Review"/ OR Evaluation/ OR Caregivers/ OR (determinant* OR influen* OR factor* OR social OR socio* OR parent* OR mother* OR father* OR maternal* OR paternal* OR lifestyle* OR life-style* OR ((behav* OR habit* OR pattern* OR practic*) ADJ3 (change* OR diet* OR feeding OR food OR snack* OR intervent* OR health* OR unhealth* OR eating)) OR (environment* ADJ3 (factor* OR condition* OR home OR school)) OR adverti* OR policy OR policies OR government* OR income OR poverty OR neighbourhood OR neighborhood OR communit* OR sedentar* OR tax OR taxes OR television OR (screen ADJ3 (view* OR time)) OR Prevent* OR reduc* OR increas* OR promot* OR education OR curriculum OR program* OR polic* OR media OR television OR campaign* OR review* OR intervention* OR initiative* OR strateg* OR evaluation OR trial* OR effect* OR impact* OR ((parent OR role) ADJ3 model*) OR caregiver* OR ((access OR availab* OR quality OR supply) ADJ3 (water OR drinkwater))).ab,ti.) NOT (exp animals/ NOT humans/) NOT (Alcohol Drinking Patterns/ OR ((alcohol OR binge) ADJ3 (drinking OR consum*)).ab,ti.) NOT (letter* OR news OR comment* OR editorial* OR congres* OR abstract* OR book* OR chapter* OR dissertation abstract*).pt. AND english.la.

**CINAHL EBSCOhost 4286**

(MH Beverages OR MH Carbonated Beverages+ OR MH Water OR MH Fruit Juices+ OR MH Tea OR MH Energy Drinks+ OR MH Sports Drinks+ OR (TI ((water OR tapwater OR fluid* OR beverage* OR liquid*) N1 (drinking)) OR ((water OR fluid* OR beverage* OR liquid* OR juice OR drink* OR tea) N5 (intake* OR consum*)) OR ((carbonat* OR fizzy OR soda OR seltzer OR co2 OR co-2 OR bubble OR sugar* OR nonsugar* OR sweet* OR nonalcohol* OR non-alcohol* OR energy OR soft OR fruit OR sucralose* OR neotame* OR acesulfame* OR saccharin* OR sport*) N2 (beverage* OR water OR drink*)) OR fruit juice* OR soda OR lemonade OR (bottle* N2 water)) OR AB (((water OR tapwater OR fluid* OR beverage* OR liquid*) N1 (drinking)) OR ((water OR fluid* OR beverage* OR liquid* OR juice OR drink* OR tea) N5 (intake* OR consum*)) OR ((carbonat* OR fizzy OR soda OR seltzer OR co2 OR co-2 OR bubble OR sugar* OR nonsugar* OR sweet* OR nonalcohol* OR non-alcohol* OR energy OR soft OR fruit OR sucralose* OR neotame* OR acesulfame* OR saccharin* OR sport*) N2 (beverage* OR water OR drink*)) OR fruit juice* OR soda OR lemonade OR (bottle* N2 water))) AND (MH Child+ OR MH Infant+ OR MH Adolescence+ OR MH "Child Behavior+" OR MH "Parent Child Relations+" OR MH "Pediatrics+" OR MH "Child Nutritional Physiology+" OR MH "Child Rearing+" OR MH "Child Psychology" OR TI (child* OR toddler* OR pre-school* OR preschool* OR schoolchild* OR primary-school* OR kindergar*) OR AB (child* OR toddler* OR pre-school* OR preschool* OR schoolchild* OR primary-school* OR kindergar*)) AND (MH Risk Factors+ OR MH Social Determinants of Health+ OR MH Parents+ OR MH Life Style+ OR MH Parent-Child Relations+ OR MH Health Policy+ OR MH Government+ OR MH Family Relations+ OR MH Advertising OR MH Television OR MH Eating Behavior+ OR MH Demography+ OR MH Taxes OR MH Preventive Health Care+ OR MH Health Promotion+ OR MH Health Education+ OR MH Evaluation Research OR MH Caregivers OR TI (determinant* OR influen* OR factor* OR social OR socio* OR parent* OR mother* OR father* OR maternal* OR paternal* OR lifestyle* OR life-style* OR ((behav* OR habit* OR pattern* OR practic*) N2 (change* OR diet* OR feeding OR food OR snack* OR intervent* OR health* OR unhealth* OR eating)) OR (environment* N2 (factor* OR condition* OR home OR school)) OR adverti* OR policy OR policies OR government* OR income OR poverty OR neighbourhood OR neighborhood OR communit* OR sedentar* OR tax OR taxes OR television OR (screen N2 (view* OR time)) OR Prevent* OR reduc* OR increas* OR promot* OR education OR curriculum OR program* OR polic* OR media OR television OR campaign* OR review* OR intervention* OR initiative* OR strateg* OR evaluation OR trial* OR effect* OR impact* OR ((parent OR role) N2 model*) OR caregiver* OR ((access OR availab* OR quality OR supply) N2 (water OR drinkwater))) OR AB (determinant* OR influen* OR factor* OR social OR socio* OR parent* OR mother* OR father* OR maternal* OR paternal* OR lifestyle* OR life-style* OR ((behav* OR habit* OR pattern* OR practic*) N2 (change* OR diet* OR feeding OR food OR snack* OR intervent* OR health* OR unhealth* OR eating)) OR (environment* N2 (factor* OR condition* OR home OR school)) OR adverti* OR policy OR policies OR government* OR income OR poverty OR neighbourhood OR neighborhood OR communit* OR sedentar* OR tax OR taxes OR television OR (screen N2 (view* OR time)) OR Prevent* OR reduc* OR increas* OR promot* OR education OR curriculum OR program* OR polic* OR media OR television OR campaign* OR review* OR intervention* OR initiative* OR strateg* OR evaluation OR trial* OR effect* OR impact* OR ((parent OR role) N2 model*) OR caregiver* OR ((access OR availab* OR quality OR supply) N2 (water OR drinkwater)))) NOT (MH animals+ NOT MH humans) NOT (TI((alcohol OR binge) N2 (drinking OR consum*)) OR AB((alcohol OR binge) N2 (drinking OR consum*))) NOT PT (letter* OR news OR comment* OR editorial* OR congres* OR abstract* OR book* OR chapter* OR dissertation abstract*) AND LA (english)

**Cochrane CENTRAL 685**

((((water OR tapwater OR fluid* OR beverage* OR liquid*) NEXT/1 (drinking)) OR ((water OR fluid* OR beverage* OR liquid* OR juice OR drink* OR tea) NEAR/6 (intake* OR consum*)) OR ((carbonat* OR fizzy OR soda OR seltzer OR co2 OR co-2 OR bubble OR sugar* OR nonsugar* OR sweet* OR nonalcohol* OR non-alcohol* OR energy OR soft OR fruit OR sucralose* OR neotame* OR acesulfame* OR saccharin* OR sport*) NEAR/3 (beverage* OR water OR drink*)) OR 'fruit juice*' OR soda OR lemonade OR (bottle* NEAR/3 water)):ab,ti) AND ((child* OR toddler* OR pre-school* OR preschool* OR schoolchild* OR primary-school* OR elementary-school* OR kindergar*):ab,ti)

**Web of science 8787**

TS=(((((water OR tapwater OR fluid* OR beverage* OR liquid*) NEAR/1 (drinking)) OR ((water OR fluid* OR beverage* OR liquid* OR juice OR drink* OR tea) NEAR/5 (intake* OR consum*)) OR ((carbonat* OR fizzy OR soda OR seltzer OR co2 OR co-2 OR bubble OR sugar* OR nonsugar* OR sweet* OR nonalcohol* OR non-alcohol* OR energy OR soft OR fruit OR sucralose* OR neotame* OR acesulfame* OR saccharin* OR sport*) NEAR/2 (beverage* OR water OR drink*)) OR "fruit juice*" OR soda OR lemonade OR (bottle* NEAR/2 water))) AND ((child* OR toddler* OR pre-school* OR preschool* OR schoolchild* OR primary-school* OR elementary-school* OR kindergar*)) AND ((determinant* OR influen* OR factor* OR social OR socio* OR parent* OR mother* OR father* OR maternal* OR paternal* OR lifestyle* OR life-style* OR ((behav* OR habit* OR pattern* OR practic*) NEAR/2 (change* OR diet* OR feeding OR food OR snack* OR intervent* OR health* OR unhealth* OR eating)) OR (environment* NEAR/2 (factor* OR condition* OR home OR school)) OR adverti* OR policy OR policies OR government* OR income OR poverty OR neighbourhood OR neighborhood OR communit* OR sedentar* OR tax OR taxes OR television OR (screen NEAR/2 (view* OR time)) OR Prevent* OR reduc* OR increas* OR promot* OR education OR curriculum OR program* OR polic* OR media OR television OR campaign* OR review* OR intervention* OR initiative* OR strateg* OR evaluation OR trial* OR effect* OR impact* OR ((parent OR role) NEAR/2 model*) OR caregiver* OR ((access OR availab* OR quality OR supply) NEAR/2 (water OR drinkwater))))) AND DT=(article) AND LA=(english)

**Google scholar 200**

"water Drinking|consumption|intake" child|children determinants|determinant|influence|factors
