# Supplementary material for: Factors associated with water consumption among children: a systematic review
Source: Int J Behav Nutr Phys Act. 2019 Aug 13;16:64. doi: 10.1186/s12966-019-0827-0 (PMC6693220; doi:10.1186/s12966-019-0827-0)
Supplement: Supplementary file 2 — ROBINS-I risk of bias protocol specified for the review on factors associated with water consumption among children. (DOCX 25 kb) [file 12966_2019_827_MOESM2_ESM.docx]

| **ROBINS-I risk of bias protocol specified for the review on factors associated with water consumption among children.** | | | | | | |
| --- | --- | --- | --- | --- | --- | --- |
|  | *Ask yourself these questions:* | **Low bias** | **Moderate bias** | **Serious bias** | **Critical bias** | **No info** |
| **1.Bias due to confounding** | *Is there potential for confounding of the effect of exposure in this study?*    *Did the authors use an appropriate analysis method that adjusted for all the critically important confounding areas e.g. in multivariable regression/stratification?*  *Were confounding areas that were adjusted for measured validly and reliably by the variables available in this study?* | No or low risk of confounding expected. | (i) Confounding expected, all known important confounding domains (sex, age (when >1 year range and not stratified) appropriately measured and controlled for;  *and*  (ii) Reliability and validity of measurement of important confounding domains were sufficient, such that we do not expect serious residual confounding. | (i) At least one known important domain was not appropriately measured, or not controlled for;  *or*  (ii) Reliability or validity of measurement of an important domain was low enough that we expect serious residual confounding. | (i) Confounding inherently not controllable  *or*  (ii) The use of negative controls strongly suggests unmeasured confounding. | No info |
| **2.Bias in selection of participants into the study** | *Did they use selection criteria (for the study or analysis) that might change the association between exposure and outcome?*  *Were the post-exposure variables that influenced selection associated with exposure, by the outcome or a cause of the outcome?*  *Were adjustment techniques used that are likely to correct for the presence of selection biases?* | (i) Selection into the study was not at all related to exposure and outcome. | (i) Selection into the study may have been related to exposure and outcome;  *and* The authors used appropriate methods to adjust for the selection bias. | (i) Selection into the study was related (but not very strongly) to exposure and outcome;  *and* This could not be adjusted for in analyses. | (i) Selection into the study was very strongly related to exposure and outcome;  *and* This could not be adjusted for in analyses. | No info |
| **3.Bias in classification of exposures** | *Is exposure status well defined and measured e.g. validated instrument (including methods used to input data)?*  *Did entry into the study begin with start of the exposure?*  *Could classification of exposure status have been affected by knowledge of the outcome or risk of the outcome?* | Measurement was objective.  Reliability and validity of measurement of exposure was high. | Measurement was subjective. Reliability and validity of measurement of exposure was moderate. | Measurement was subjective. Reliability and validity of measurement of exposure was low. | Measurement was subjective. Reliability and validity of measurement of exposure was very low. | No info |
| **4.Bias due to departures from intended exposures**  *(only for longitudinal studies)* | *Is there concern that changes in exposure status occurred among participants?*  *Were adjustment techniques used that are likely to correct for these issues?* | No concern that changes in exposure status occurred among participants | Some concern that changes in exposure status occurred among participants  Adjustment techniques used likely to corrected for these issues | Some concern that changes in exposure status occurred among participants  Adjustment techniques used could did not correct for these issues | High concern that changes in exposure status occurred among participants  Adjustment techniques used could did not correct for these issues | No info |
| **5.Bias due to missing data** | *Were there missing outcome, exposure or confounding data?*  *Are the proportion of participants and reasons for missing data similar across exposures?*  *Were appropriate statistical methods used to account for missing data?* | (i) Data were reasonably complete (<5% missings);  *or*  (ii) (data of) Persons in and excluded had similar characteristics.  *or*  (iii) The analysis addressed missing data and is likely to have removed any risk of bias (e.g. weighting of data or multiple imputation). | (i) (data of) Persons in and excluded differed slightly.  *and*  (ii) The analysis is unlikely to have removed the risk of bias arising from the missing data. | (i) (data of) Persons in and excluded differed substantially.  *and*  (ii) The analysis is unlikely/unable to have removed the risk of bias arising from the missing data; | (i) (data of) Persons in and excluded differed critically.  *and*  (ii) Missing data were not, or could not, be addressed through appropriate analysis. | No info |
| **6.Bias in measurement of outcomes** | *Did the authors use objective outcome measures?*  *Could the outcome measure have been influenced by knowledge of the exposure received?*  *Was the outcome measure sensitive and valid?*  *Was it accurately measured?* | Measurement was objective.  Reliability and validity of measurement of outcome was high.   - Prospective multiday weighed food record | Measurement was subjective. Reliability and validity of measurement of outcome was moderate.  Estimates by non-parent observer  Prospective multiday non-weighed food record  multiday 24-hr recall | Measurement was subjective. Reliability and validity of measurement of outcome was low.   - FFQ - Single day 24h recall   Prospective single day non-weighed food record | Measurement was subjective. Reliability and validity of measurement of outcome was very low.   - Specific critical problem with measurement | No info |
| **7.Bias in selection of the reported result** | *Is the reported effect estimate likely to be selected, on the basis of the results, from...?*  *... multiple outcome measurements within the outcome domain?*  *... multiple analyses of the exposure-outcome relationship?*  *... different subgroups?*  *Examples bias in analysis: unadjusted and adjusted models; a continuously scaled outcome converted to categorical data with different cut-points, different sets of covariates used for adjustment;* | There is clear evidence that all reported results correspond to all intended outcomes, analyses and sub-cohorts. The study and specific analyses are registered in a protocol paper. | (i) The aims, outcome measurements and analyses are clearly defined and consistent;  *and*  (ii) There is no indication of selection of the reported analysis from among multiple analyses  *and*  (iii) There is no indication of selection of the cohort or subgroups for analysis and reporting on the basis of the results. | (i) Outcomes are defined in different ways in the methods and results sections;  *or*  (ii) There is a high risk of selective reporting from among multiple analyses,  *or*  (iii) The cohort or subgroup is selected from a larger study for analysis and appears to be reported on the basis of the results. | (i) There is evidence or strong suspicion of selective reporting of results;  *and*  (ii) The unreported results are likely to be substantially different from the reported results. | No info |
| **Overall risk of bias** | *The lowest rating determines overall risk of bias. E.g. if you scored the study ‘moderate’ in 6 domains but ‘serious’ in 1 domain the overall risk of bias is serious.*  *No info: there is no clear indication that the study is at serious or critical risk of bias and there is a lack of information in one or more key domains of bias* | low | moderate | serious | critical | No info |
