# Supplementary material for: Factors associated with water consumption among children: a systematic review
Source: Int J Behav Nutr Phys Act. 2019 Aug 13;16:64. doi: 10.1186/s12966-019-0827-0 (PMC6693220; doi:10.1186/s12966-019-0827-0)
Supplement: Supplementary file 3 — Characteristics, associations and risk of bias of studies included in the review on factors associated with water consumption among children. (DOCX 85 kb) [file 12966_2019_827_MOESM3_ESM.docx]

| **Table S1: Characteristics and associations of studies included in the review on factors associated with water consumption among children** | | | | | | | | | | | | | | | |
| --- | --- | --- | --- | --- | --- | --- | --- | --- | --- | --- | --- | --- | --- | --- | --- |
| **Cross-sectional studies** | | | | | | | | | | | | | | | |
| **1st author, year** | **Country** | | **Design** | **Population** | | **N** | **Age** | | **Outcome** | | **Measure** | | **Factor** | | **Association*** |
| **Ayala, 2008** | USA | | CS | Children and parents with Latino background from 13 schools in Southern Califonia | | 708 | 4-7 y | | Number of servings of water in a day | | FFQ | | Consuming foods at relatives/neighbors/friends (≥once/week vs <once/week) | | - |
|  |  |  |  |  |  |  |  |  |  |  |  |  | Consuming foods at fastfood/buffet/sit down (≥once/week vs <once/week) | | -- |
|  |  |  |  |  |  |  |  |  |  |  |  |  | The main type of restaurant (fastfood/buffet/sit down) | | / |
| **Barraj, 2009** | USA | | CS | Representative sample of children and parents of the US population | | 549 | 3-5 y and 6-12 y | | Water consumption in ounces/day | | 7 day dietary record | | Age: 6-12 group vs 3-5 group | | ++ |
|  |  |  |  |  |  |  |  |  |  |  |  |  | Season (summer) vs winter | | ++ |
| **Beltrán-Aguilar, 2015** | USA | | CS | Representative sample of children and parents of the US population (NHANES). | | 4104 | 1-10 y | | Tap/bottled water consumption in ml/kg/day. | | 1 day 24 hour dietary recall | | Temperature | | ++ |
|  |  |  |  |  |  |  |  |  |  |  |  |  | Age | | -- |
|  |  |  |  |  |  |  |  |  |  |  |  |  | Sex (female) | | + |
|  |  |  |  |  |  |  |  |  |  |  |  |  | Ethnicity (non-white) | | + |
|  |  |  |  |  |  |  |  |  |  |  |  |  | Poverty status (higher income) | | / |
| **Bougatsas, 2018** | Greece | | CS | Healthy children from Athens and surrounding suburban areas | | 210 | 8-14 y; mean=11 y | | Water consumption in ml/day | | 2 day dietary record | | Sex (female) | | / |
| **Campbell, 2010** | Australia | | CS | Children and mothers from health centres and kindergartens in a rural region of Victoria | | 80 | 5 y | | Number of servings of water in a day (0-6 or more) | | 1 day 24 hour dietary recall | | maternal self-efficacy promoting healthy eating in child (fruit, vegetables, plain water, enough food and a wide range of foods over the next year) | | ++ |
|  |  |  |  |  |  |  |  |  |  |  |  |  | maternal self-efficacy limiting non-core foods for child (sweet snacks, confectionary, lollies or ice cream; potato crisps/salty snack foods and biscuits; soft drink, cordial and other sweetened drinks; energy dense foods/drinks) | | + |
| **Campos, 2009** | Guatemala | | CS | Children of 12 schools in the urban zones of Quetzaltenango City | | 449 | 9-12 y | | Frequency of consumption and ml of water consumption in a day | | 1 day 24 hour dietary recall | | Gender (Female) | | / |
|  |  |  |  |  |  |  |  |  |  |  |  |  | SES (public school vs private school) | | / |
|  |  |  |  |  |  |  |  |  |  |  |  |  | Meal time (lunch vs dinner, breakfast and snacks) | | ++ |
| **Cardon, 2016** | Belgium, Bulgaria, Germany, Greece, Poland, Spain | | CS | Parents and children from kindergartens, daycare centres or preschool settings in six countries (Toybox study) | | 3301 | 4-6 y | | Water consumption in ml/day | | FFQ | | overweight | | ++ |
| **Condon, 2009** | USA | | CS | Representative sample of parents and children from public schools | | 2314 | 5-13 y | | Bottled water consumption at breakfast/lunch (yes/no) | | 1 day 24 hour dietary recall | | National school lunch program participation | | lunch: -- primary&middle school. breakfast / primary -- middle school. |
| **Coppinger, 2013** | UK | | CS | Children from primary and secondary schools in south-west London | | 248 | 9-13 y | | Water consumption in ml/day | | 3 day dietary record | | age group (9-10 vs 11-13) | | / |
|  |  |  |  |  |  |  |  |  |  |  |  |  | Gender | | / |
| **Cullen, 2004** | USA | | CS | African-American girls and parents who attended two field centers | | 95 |  | | Number of servings of water in a day | | 2 day 24 hour dietary recall | | Parent experiences barriers preparing, serving and consuming low-fat food | | -- |
|  |  |  |  |  |  |  |  |  |  |  |  |  | Child has higher preference for water | | ++ |
|  |  |  |  |  |  |  |  |  |  |  |  |  | Child has higher preference for SSBs (soft drinks, fruit drinks, sports drinks, sweetened tea/coffee/water) | | - |
| **Cunningham, 2012** | USA | | CS | Stratified, random sample of Oregon resident mothers and their children (PRAMS-2) | | 1522 | 2 y | | Number of days in a week consuming water: 0–1 days/week versus 2–3 days/week and 4–7 days/week | | FFQ | | SES (food insecurity) | | - |
| **Dai, 2014** | Taiwan | | CS | Students in five public elementary schools in Taipei | | 222 | 10-13 y; mean=11.9 y | | Number of glasses of water in a day | | 1 day 24 hour dietary recall | | Self-efficacy for drinking water | | ++ |
| **Danyliw, 2011** | Canada | | CS | Representative sample of children and parents in Canada (Canadian Community Health Survey) | | 10038 | 2-5 and 6-11 y | | Water (Tap and bottled water, club soda, sparkling water) consumption in gramms/day | | 1 day 24 hour dietary recall | | Beverage cluster (soft drinks, fruit drinks, fruit juice, milk) | | / |
| **De Craemer, 2015** | Belgium, Bulgaria, Germany, Greece, Poland, Spain | | CS | Parents and children from kindergartens, daycare centres or preschool settings in six countries (Toybox study) | | 4045 | 4-6 y | | Water consumption in ml/day | | FFQ | | country | | highest-lowest= Spain, Bulgaria, Greece, Germany, Belgium, Poland |
| **Dodd, 2013** | USA | | CS | Representative sample of US public school children (SNDA-III) | | 732 | 6-12 y | | Any bottled water consumption (yes/no) | | 1 day 24 hour dietary recall | | Race | | / |
|  |  |  |  |  |  |  |  |  |  |  |  |  | Overweight | | / |
| **Drewnowski, 2013** | USA | | CS | Representative sample of children and parents in US (NHANES) | | 4766 | 4-13 y | | Tap/bottled water consumption in ml/day | | 2 day 24 hour dietary recall | | Age group (9-13 vs 4-8) | | ++ |
|  |  |  |  |  |  |  |  |  |  |  |  |  | Sex (female) | | + |
|  |  |  |  |  |  |  |  |  |  |  |  |  | Income (lower) | | - |
|  |  |  |  |  |  |  |  |  |  |  |  |  | Race/ethnicity (non-white) | | -- |
| **Dubuisson, 2015** | France | | CS | Representative samples of children and parents living in mainland France (INCA2) | | 463 | pre-school aged mean=7.3 y | | Number of times consuming water in a week at lunch and in total | | 7 day dietary record | | School lunch attendance (never/occasional/regular) | | / |
|  |  |  |  |  |  |  |  |  |  |  |  |  | Consumption at school vs other location | | - |
| **Ebenegger, 2011** | Switzerland | | CS | Kindergarten children and parents from areas with a high migrant prevalence in two Swiss cantons | | 542 | mean=5.1 y | | Number of times consuming water in a day | | FFQ | | Migrant status at least 1 parent (migrant) | | / |
|  |  |  |  |  |  |  |  |  |  |  |  |  | Education level at least 1 parent (low) | | -- |
| **Elmore, 2013** | USA | | CS | African-American children from three public schools in two large Midwestern cities | | 222 | 9-13 y; mean=10.8 y | | Number of glasses of water in a day | | 1 day 24 hour dietary recall | | Self-control for drinking water | | ++ |
|  |  |  |  |  |  |  |  |  |  |  |  |  | Self-efficacy for drinking water | | ++ |
| **Evans, 2016** | UK | | CS | Parents and children from 54 schools across England | | 2709 | 6-8 y | | Consuming of water in a day (yes/no) | | 1 day dietary record | | Type lunch (school meal vs packed lunch) | | ++ |
| **Feferbaum, 2012** | Brazil | | CS | Representative sample of parents and children from Brazilian urban areas | | 837 | 3-6 and 7-10 y | | Water consumption in L/day | | 4 day dietary record | | age group (7-10 vs 3-7 years) | | ++ |
| **Fernández-Alvira, 2014** | Spain | | CS | Representative sample of parents and children of Spain | | 238 | 3-17 y; mean=9.2 y | | Water consumption (tap water, filtered tap water, natural mineral water, sparkling natural mineral water, flavoured water, fountain water) in ml/day | | 7 day 24 hour dietary recall | | Sex (female) | | - |
|  |  |  |  |  |  |  |  |  |  |  |  |  | Age (7-11 vs 3-6) | | + |
|  |  |  |  |  |  |  |  |  |  |  |  |  | intake moment (during the meal vs outside the meal) | | ++ |
| **Fernández-Alvira, 2013** | Italy, Estonia, Cyprus, Belgium, Sweden, Hungary, Germany, Spain | | CS | Parents and children from primary schools and pre-schools of selected regions in 8 countries (IDEFICS) | | 14426 | 2-9 y | | Number of times consuming water in a week (divided into tertiles of consumption; high versus medium and low). | | FFQ | | Education level (low) | | overall -- stratified by country -- Belgium, Hungary, Italy and / Estonia, Cyprus, Sweden, Germany, Spain |
| **Franckle, 2015** | USA | | CS | Children of public schools in two Massachusetts communities; low-income and with large Hispanic populations | | 1870 | mean=10.6 y | | Number of times consuming water in a day | | 1 day 24 hour dietary recall | | Sleep duration (insufficient) | | - |
| **Guelinckx, 2015** | Mexico, Brazil, Argentina, Uruguay, Spain, France, Belgium, UK, Poland, Turkey, Iran, China, Indonesia | | CS | Parents and children from schools in 11 countries | | 3611 | 4-9 y | | Water (Still water, unflavored sparkling water, tap/filtered/boiled water) consumption in ml/day | | 7 day dietary record | | Country | | highest-lowest boys: Indonesia, Spain, Turkey, Uruguay, Iran, China, France, Brazil, UK, Mexico, Belgium, Argentina, Poland. Girls: Indonesia, Uruguay, Turkey, Spain, China, Iran, Brazil, UK, Argentina, Mexico, Belgium, Poland. |
| **Hoffmann, 2018** | USA | | CS | Mothers and children of a two-parent household recruited through Amazon’s Mechanical Turk in US | | 192 | 7-11 y | | Frequency of consumption of water on weekday and weekend day | | FFQ | | weekday vs weekend | | ++ |
| **Inhulsen, 2017** | Netherlands | | CS | Children and parents from municipal registry of a North-western municipality | | 5926 | 3-7 y | | Number of glasses of water in a day (range 0.5-6) | | FFQ | | Parental feeding practice (control over eating) | | - |
|  |  |  |  |  |  |  |  |  |  |  |  |  | Parental feeding practice (emotional feeding) | | - |
|  |  |  |  |  |  |  |  |  |  |  |  |  | Parental feeding practice (encouragement for eating) | | ++ |
|  |  |  |  |  |  |  |  |  |  |  |  |  | Parental feeding practice (instrumental feeding) | | -- |
| **Jomaa, 2016** | Lebanon | | CS | Representative sample of children and parents in Lebanon | | 752 | 4-13 y | | Bottled/tap water consumption in ml/day | | FFQ | | age group(9-13 vs 4-8 years) | | ++ |
|  |  |  |  |  |  |  |  |  |  |  |  |  | Sex (female) | | -- |
|  |  |  |  |  |  |  |  |  |  |  |  |  | Education level (lower) | | / |
|  |  |  |  |  |  |  |  |  |  |  |  |  | SES; employment status (unemployed) | | + |
|  |  |  |  |  |  |  |  |  |  |  |  |  | Family income (lower) | | / |
|  |  |  |  |  |  |  |  |  |  |  |  |  | Crowding index (≥2) | | - |
|  |  |  |  |  |  |  |  |  |  |  |  |  | BMI (obese/overweight/normal weight) | | + |
|  |  |  |  |  |  |  |  |  |  |  |  |  | Waist to height ratio (≥0.5) | | - |
|  |  |  |  |  |  |  |  |  |  |  |  |  | Level of physical activity (active vs inactive) | | + |
| **Kakietek, 2014** | USA | | CS | Children of 106 group child centers in low-income areas in New York City | | 636 | 3-4 y | | Consumption of water (yes/no) with any meal or snack during the day in child care | | Observation by researcher | | Child care center compliance with NYC regulations and beverage consumption | | - |
|  |  |  |  |  |  |  |  |  |  |  |  |  | Child and Adult care food program (nutritious meals and snacks to low income families) | | -- |
|  |  |  |  |  |  |  |  |  |  |  |  |  | Head start (a comprehensive developmental program for low income families) | | ++ |
|  |  |  |  |  |  |  |  |  |  |  |  |  | Center in a District Public Health Office catchment area (program that targets resources to high-need neighborhoods) | | ++ |
|  |  |  |  |  |  |  |  |  |  |  |  |  | Classroom size | | - |
|  |  |  |  |  |  |  |  |  |  |  |  |  | Operating hours | | ++ |
|  |  |  |  |  |  |  |  |  |  |  |  |  | Student-teacher ratio | | - |
|  |  |  |  |  |  |  |  |  |  |  |  |  | Teaching staff tunrover | | ++ |
|  |  |  |  |  |  |  |  |  |  |  |  |  | Center participated in Eat Well Play Hard (teaches staff and children about nutrition and physical activity) | | + |
|  |  |  |  |  |  |  |  |  |  |  |  |  | No. of nutrition-related training programs other than Eat Well Play Hard in which the center participated | | + |
|  |  |  |  |  |  |  |  |  |  |  |  |  | No. of staff in the center who participated in Training of Teachers | | + |
|  |  |  |  |  |  |  |  |  |  |  |  |  | No. of staff in the classroom who participated in Training of Teachers Eat Well Play Hard (teaches staff and children about nutrition and physical activity) | | - |
|  |  |  |  |  |  |  |  |  |  |  |  |  | No. of meals the child had during the day of observation | | ++ |
| **Kant, 2010** | USA | | CS | Representative sample of children and their parents in US (NHANES) | | 1870 | 2-5 and 6-11 y | | Consumption of water (tap, from cooler or fountain, spring water, noncarbonated bottled) gramms/day | | 1 day 24 hour dietary recall | | Moisture in beverages (100 g) | | -- (2-5y) -- (6-11y) |
|  |  |  |  |  |  |  |  |  |  |  |  |  | Energy (100 kcal) | | - (2-5y) - (6-11y) |
|  |  |  |  |  |  |  |  |  |  |  |  |  | Amount of foods (100 g) | | -- (2-5y) ++ (6-11y) |
|  |  |  |  |  |  |  |  |  |  |  |  |  | Fat (5 g) | | + (2-5y) + (6-11y) |
|  |  |  |  |  |  |  |  |  |  |  |  |  | Protein (5 g) | | + (2-5y) + (6-11y) |
|  |  |  |  |  |  |  |  |  |  |  |  |  | Carbohydrate (10 g) | | - (2-5y) + (6-11y) |
|  |  |  |  |  |  |  |  |  |  |  |  |  | Total sugars (5 g) | | -- (2-5y) -- (6-11y) |
|  |  |  |  |  |  |  |  |  |  |  |  |  | Fiber (5 g) | | + (2-5y) ++ (6-11y) |
|  |  |  |  |  |  |  |  |  |  |  |  |  | Sodium (100 mg) | | + (2-5y) + (6-11y) |
|  |  |  |  |  |  |  |  |  |  |  |  |  | Number of eating occasions | | + (2-5y) + (6-11y) |
|  |  |  |  |  |  |  |  |  |  |  |  |  | Mentioned a snack in the recall | | ++ (2-5y) - (6-11y) |
|  |  |  |  |  |  |  |  |  |  |  |  |  | Mentioned breakfast in the recall | | ++ (2-5y) - (6-11y) |
| **Kaushik, 2007** | UK | | CS | Children from six urban Southampton primary schools | | 298 | 6-7 and 9-10 y | | Water (from 'fluid containers' and water fountain) consumption during school day in ml | | Weighed 1 day fluid intake recorded by observer | | Free access to drinking water in classroom and allowed having water bottle on desk (vs prohibited access or limited access) | | ++ |
| **Lioret, 2010** | France | | CS | Representative sample of children and their parents in France (INCA) | | 574 | 3-10 y | | Water (Plain and carbonated mineral waters, tap water, spring water) in ml/day | | 7 day dietary record | | Sex (female) | | -- |
| **Lora, 2016** | USA | | CS | African-American and Hispanic fathers and their children in Oklahoma City | | 110 | 2-5 y | | Water consumption in fluid ounces/day | | FFQ | | Difficulty in child feeding (perceived barriers) | | -(AA) -(H) |
|  |  |  |  |  |  |  |  |  |  |  |  |  | Desire to drink any beverage child | | -(AA) -(H) |
|  |  |  |  |  |  |  |  |  |  |  |  |  | Concern about the child being overweight | | +(AA) +(H) |
|  |  |  |  |  |  |  |  |  |  |  |  |  | Use of food to calm the child (emotional) | | -(AA) +(H) |
|  |  |  |  |  |  |  |  |  |  |  |  |  | Concern about the child being underweight | | -(AA) -(H) |
|  |  |  |  |  |  |  |  |  |  |  |  |  | Use of food as reward (instrumental) | | -(AA) -(H) |
| **Maffeis, 2016** | Italy | | CS | Parents and children from a large primary school district in the main residential area of Verona | | 175 | 7-11 y | | water consumption in ml/day | | 3 day weighed dietary record | | Obese vs normal weight | | + |
| **Makkes, 2011** | Guatemala | | CS | Parents and children from public and private schools from urban Quetzaltenango | | 356 | 8-10 y | | Water consumption (yes/no) and in ml/day | | 1 day dietary record | | SES (public school vs private school) | | - |
| **Mantziki, 2017** | Belgium, France, Greece, Portugal, Romania, Netherland, Bulgaria | | CS | Families with children from schools in communities 7 countries (EPHE; EPODE methodology) | | 1187 | 6-8 y | | High daily water (artesian well, spring, mineral or sparkling) consumption; dichotomized on basis of median low (<5-6 times a day) and high (>5-6 times a day) | | FFQ | | Frequency of SD consumption (low) | | ++ |
|  |  |  |  |  |  |  |  |  |  |  |  |  | Frequency of FJ consumption (low) | | / |
|  |  |  |  |  |  |  |  |  |  |  |  |  | Parent monitoring amount (monitoring) | | -- moderate - low (FJ) -- moderate / low (SD) |
|  |  |  |  |  |  |  |  |  |  |  |  |  | Allowing consumption if asked (restriction) | | + moderate + low (FJ) ++ moderate ++ low (SD) |
|  |  | |  |  | |  |  | |  | |  | | Allowing whenever child wants (restriction) | | + moderate + low (FJ) + moderate ++ low (SD) |
|  |  |  |  |  |  |  |  |  |  |  |  |  | Negotiating amount allowed (negotiating) | | - moderate - low (FJ) - moderate -- low (SD) |
|  |  |  |  |  |  |  |  |  |  |  |  |  | Communicate health belief (telling child that FJ/SD are not good for them) | | - moderate / low (FJ) - moderate -- low (SD) |
|  |  |  |  |  |  |  |  |  |  |  |  |  | Communicate health belief (telling child that FJ/SD make them fat) | | -- moderate - low (FJ) - moderate -- low (SD) |
|  |  |  |  |  |  |  |  |  |  |  |  |  | Avoid negative modelling | | - moderate / low (FJ) - moderate -- low (SD) |
|  |  |  |  |  |  |  |  |  |  |  |  |  | Parental efficacy to manage child's intake of FJ/SD | | + moderate ++ low (FJ) + moderate ++ low (SD) |
|  |  |  |  |  |  |  |  |  |  |  |  |  | Rewarding/comforting practice (emotional) | | + moderate + low (FJ) - moderate - low (SD) |
|  |  |  |  |  |  |  |  |  |  |  |  |  | Drinking sweet drinks together with child (modelling) | | + moderate - low (FJ) -- moderate + low (SD) |
|  |  |  |  |  |  |  |  |  |  |  |  |  | Home availability FJ/SD | | - moderate + low (FJ) + moderate ++ low (SD) |
| **Mantziki, 2015** | Belgium, France, Greece, Portugal, Romania, Netherlands, Bulgaria | | CS | Families with children from schools in communities 7 countries (EPHE; EPODE methodology) | | 1187 | 6-8 y | | Water (artesian well, spring, mineral or sparkling) consumption in number of times per day | | FFQ | | Education level mother (lower) | | / |
| **Milla Tobarra, 2018** | Spain | | CS | Parents and children from 20 public schools in Cuenca province | | 182 | 9-11 y | | Water consumption in ml/day | | 1 day 24 hour dietary recall | | SES parents (index based on education level and occupation parents, divided into low, mid, high) | | / |
| **Murnan, 2006** | China | | CS | Children from three primary schools in Bejing | | 282 | 9-12 y | | Number of glasses of water in a day | | 1 day 24 hour dietary recall | | Self-efficacy to drink water | | ++ |
|  |  |  |  |  |  |  |  |  |  |  |  |  | Sex (unclear male/female) | | -- |
|  |  |  |  |  |  |  |  |  |  |  |  |  | Number of times taught in school about physical activity (knowledge) | | ++ |
| **Papandreou, 2013** | Greece | | CS | Parents and children from ten schools in Thessaloniki city | | 607 | 7-15 y | | Water consumption in ml/day | | 3 day 24 hour dietary recall | | Sex (female) | | -- |
|  |  |  |  |  |  |  |  |  |  |  |  |  | BMI (obese vs normal) | | ++ |
| **Parsons, 1999** | United Kingdom | | CS | First- and second-generation Pakistani mothers and children in Bradford | | 226 | 3 y | | Water consumption at different meals (breakfast, lunch, dinner, snack) yes/no | | 1 day 24 hour dietary recall | | Generation of immigration (first vs second) | | ++ |
| **Patel, 2014** | USA | | CS | Children in 9 California middle schools in a predominantly Latino district (sNaX trial) | | 2873 | mean=12.3 y | | Number of glasses of tap/bottled water in a day | | 1 day 24 hour dietary recall | | Intentions to drink water the next day at school | | ++ |
|  |  |  |  |  |  |  |  |  |  |  |  |  | Sex (Female) | | -- |
|  |  |  |  |  |  |  |  |  |  |  |  |  | Age | | + |
|  |  |  |  |  |  |  |  |  |  |  |  |  | Ethnicity (Black vs Latino) | | + |
|  |  |  |  |  |  |  |  |  |  |  |  |  | Ethnicity (other vs Latino) | | ++ |
|  |  |  |  |  |  |  |  |  |  |  |  |  | Speak English at home | | -- |
|  |  |  |  |  |  |  |  |  |  |  |  |  | US born | | - |
|  |  |  |  |  |  |  |  |  |  |  |  |  | Eligibility for free and reduced lunch (lower SES) | | - |
| **Patel, 2013** | USA | | CS | Nationally representative sample of children and parents in the US (NHANES) | | 4293 | 1-11 y | | Any tap water consumption (yes/no) | | 1 day 24 hour dietary recall | | age (older) | | ++ |
| **Petter, 1995** | United Kingdom | | CS | Parents and (pre-)school children in and around Southamption | | 105 | 2-7 y | | Number of times consumed water in two days | | 2 day dietary record | | preshool vs schoolaged children (age) | | / |
| **Piernas, 2014** | Mexico | | CS | Representative sample of children and parents in Mexico (NHNS) | | 3932 | 4-8 and 9-13 y | | Water consumption in ml/day | | 1 day 24 hour dietary recall | | age (older) | | ++ |
|  |  |  |  |  |  |  |  |  |  |  |  |  | Sex (female) | | -- (age 4-8) - (age 9-13) |
| **Pinket, 2016a** | Belgium, Bulgaria, Germany, Greece, Poland, Spain | | CS | Children in kindergartens, daycare centres or preschool settings in six European countries (ToyBox study) | | 6776 | 3-5 y | | Tap/bottled water consumption in ml/day | | FFQ | | Availability SD/prepacked juice always and/or on table during meal | | -- |
|  |  |  |  |  |  |  |  |  |  |  |  |  | Availability water always and/or on table during meal | | ++ |
|  |  |  |  |  |  |  |  |  |  |  |  |  | Permissiveness towards SD/prepacked juices (opposite restriction parenting) | | -- |
|  |  |  |  |  |  |  |  |  |  |  |  |  | Avoiding negative modelling SD/prepacked juice | | - |
|  |  | |  |  | |  |  | |  | |  | | Awareness negative advice SD/prepacked juice consumption (knowledge) | | - |
|  |  |  |  |  |  |  |  |  |  |  |  |  | Encouragement to drink water | | ++ |
|  |  |  |  |  |  |  |  |  |  |  |  |  | Rewarding with SD/prepacked juices when child upset (emotional) | | ++ |
|  |  |  |  |  |  |  |  |  |  |  |  |  | Lack of self-efficacy giving child water when they want SD/prepacked juice | | -- |
| **Pinket, 2016b** | Belgium, Bulgaria, Germany, Greece, Poland, Spain | | CS | Children in kindergartens, daycare centres or preschool settings in six European countries (ToyBox study) | | 7051 | 3-5 y | | Tap/bottled water consumption in ml/day | | FFQ | | Sex (female) | | -- |
|  |  |  |  |  |  |  |  |  |  |  |  |  | Education level mother (low) | | -- |
| **Senterre, 2014** | Belgium | | CS | Parents and children in 20 schools of 9 provinces in Belgium | | 1045 | 8-13 y | | Water consumption in ml/day | | 7 day dietary record | | Physical activity (higher) | | ++ |
| **Sharma, 2005** | USA | | CS | Children from six schools in Kentucky | | 159 | 10-12 y | | Number of glasses of water in a day | | 1 day 24 hour dietary recall | | Expectations drinking 8 or more glasses water/day | | ++ |
| **Sichieri, 2013** | Brazil | | CS | Children from 22 public schools in the metropolitan area of Rio de Janeiro | | 1134 | 10-11 y | | Number of glasses of water in a day (0-7) | | FFQ | | Sex (female) | | - |
|  |  |  |  |  |  |  |  |  |  |  |  |  | BMI (underweight/normal/overweight/obese) | | + |
|  |  |  |  |  |  |  |  |  |  |  |  |  | Sodas | | - |
|  |  |  |  |  |  |  |  |  |  |  |  |  | Guarana | | / |
|  |  |  |  |  |  |  |  |  |  |  |  |  | Fruit juice | | + |
|  |  |  |  |  |  |  |  |  |  |  |  |  | Milk | | ++ |
| **Sohn, 2001** | USA | | CS | Representative sample of children and parents in the US (NHANES) | | 7925 | 1-10 y | | Water consumption in ml/kg/day | | 1 day 24 hour dietary recall | | age (older) | | -- |
|  |  |  |  |  |  |  |  |  |  |  |  |  | Sex (female) | | - |
|  |  |  |  |  |  |  |  |  |  |  |  |  | race (not white) | | ++ (AA) + (MA) - (other) |
|  |  |  |  |  |  |  |  |  |  |  |  |  | SES (low poverty income ratio) | | ++ |
| **Terry, 2017** | Australia | | CS | Parents and children from primary schools in rural or regional North East Victoria | | 550 | 5-11 y | | Servings of water in a day | | FFQ | | temperature (higher) | | + |
|  |  |  |  |  |  |  |  |  |  |  |  |  | Plain milk | | + |
|  |  |  |  |  |  |  |  |  |  |  |  |  | Snacks | | - |
|  |  |  |  |  |  |  |  |  |  |  |  |  | Sweets | | - |
|  |  |  |  |  |  |  |  |  |  |  |  |  | Cakes | | - |
|  |  |  |  |  |  |  |  |  |  |  |  |  | Fruit juice | | - |
|  |  |  |  |  |  |  |  |  |  |  |  |  | Soft drink | | -- |
|  |  |  |  |  |  |  |  |  |  |  |  |  | Cordial (un-carbonated sugar sweetened beverage) | | -- |
|  |  |  |  |  |  |  |  |  |  |  |  |  | Fruit | | ++ |
|  |  |  |  |  |  |  |  |  |  |  |  |  | Vegetables | | ++ |
|  |  |  |  |  |  |  |  |  |  |  |  |  | SES (health care card recipients) | | -- |
| **Vereecken, 2008** | Belgium | | CS | Parents and children from 50 nursery schools in Flanders | | 1678 | 2-6 y | | Water consumption in ml/day | | FFQ | | School | | / |
| **Vieux, 2016** | France | | CS | Representative sample of children and parents in France (INCA2) | | 835 | 4-13 y | | Tap/bottled water consumption in ml/day | | 7 day dietary record | | age (older) | | ++ |
|  |  |  |  |  |  |  |  |  |  |  |  |  | Sex (female) | | -- |
|  |  |  |  |  |  |  |  |  |  |  |  |  | Family income (lower) | | / |
| **Vieux, 2017** | United Kingdom | | CS | Representative sample of children and parents in the UK (NDNS) | | 845 | 4-13 y | | Tap/bottled water consumption in ml/day | | 4 day dietary record | | age (older) | | + |
|  |  |  |  |  |  |  |  |  |  |  |  |  | Sex (female) | | + |
|  |  |  |  |  |  |  |  |  |  |  |  |  | Family income (lower) | | -- |
|  |  |  |  |  |  |  |  |  |  |  |  |  | Season | | / |
|  |  |  |  |  |  |  |  |  |  |  |  |  | BMI (overweight/obese) | | - |
|  |  |  |  |  |  |  |  |  |  |  |  |  | Ethnicity (non white) | | + |
|  |  |  |  |  |  |  |  |  |  |  |  |  | Region (south incl. London) vs central & vs Scotland/Wales/Northern Ireland | | ++ |
| **Wang, 2016** | USA | | CS | Representative sample of children and parents in the US (NHANES) | | 3647 | 4-13 y | | Water (plain tap and bottled water, carbonated water, and flavored or fortified water) consumption during morning, afternoon and evening (yes/no) | | 2 day 24 hour dietary recall | | age (older) | | / |
| **Watowicz, 2014** | USA | | CS | Representative sample of children and parents in the US (NHANES) | | 1963 | 2-4 y | | Water consumption in grams/day | | 1 day 24 hour dietary recall | | SES; WIC participation (vs low/high income) | | - |
| **Longitudinal/repeated cross-sectional studies** | | | | | | | | | | | | | | | |
| **1st author, year** | **Country** | **Design** | | **Analysis** | **Population** | | **N** | **Age** | | **Outcome** | | **Measure** | | **Factor** | **Association*** |
| **Bleich, 2017** | USA | RCS; 11 y FU | | Weighted multivariate regression | Representative sample of children and parents of the US population (NHANES) | | 9992 | 2-5 y and 6-11 y | | Any water consumption (yes/no) | | 1 day 24 hour dietary recall | | Time (more recent from 2004-2014) | ++ |
| **Cockburn, 2017** | Australia | L; 8 y FU | | Chi-squared test at 6 time points | Representative cohort of children and parents in Australia (LSAC study) | | 5107 | 2-10 y | | Water consumption in a day (yes/no) | | 1 day 24 hour dietary recall | | Age | / |
|  |  |  |  |  |  |  |  |  |  |  |  |  |  | Sex (female) | ++ (at 4,6,8,10 years) |
|  |  |  |  |  |  |  |  |  |  |  |  |  |  | region | / |
|  |  |  |  |  |  |  |  |  |  |  |  |  |  | medical condition | / |
|  |  |  |  |  |  |  |  |  |  |  |  |  |  | birth country (australia) | / |
|  |  |  |  |  |  |  |  |  |  |  |  |  |  | Language (not-English speaking) | / |
|  |  |  |  |  |  |  |  |  |  |  |  |  |  | Indigenous | -- (at 2,4,6,8,10 years) |
|  |  |  |  |  |  |  |  |  |  |  |  |  |  | SES (lower socio-economic index for areas) | -- (at 0,2,6,8 years) |
| **Haroun, 2011** | UK | RCS; 4 y FU | | Chi-squared test | Children having school lunches in a representative sample of 136 primary schools in England | | 6696 | 3-12 y | | Taking water (still or carbonated; unsweetened, unflavoured) at school lunch (Yes/No) | | Observation by field worker during lunch | | Time (2009 compared with 2005) | ++ |
| **Sichert-Hellert, 2001** | Germany | L; 14 y FU | | Mixed multivariate linear model | Mothers and their children from maternity wards (DONALD cohort study) | | 733 | 2-13 y | | Water (tap or mineral) consumption in grams/day | | 3 day weighed dietary record | | Time (more recent from 1985-1999) | ++ (for tapwater all ages) ++ (for mineral water 2-8y) + (for mineral water 9-13 y) |
| **Sleddens, 2014** | Netherlands | L; 2 y FU | | Multivariate linear regression analysis adjusted for confounding not baseline water consumption | Healthy mothers and their children and those with an alternative lifestyle (KOALA cohort study) | | 1654 | 6-8 y | | Number of glasses of water in a week | | FFQ | | BMI | ++ |
|  |  |  |  |  |  |  |  |  |  |  |  |  |  | Instrumental feeding | - |
|  |  |  |  |  |  |  |  |  |  |  |  |  |  | Emotional feeding | - |
|  |  |  |  |  |  |  |  |  |  |  |  |  |  | Control | - |
|  |  |  |  |  |  |  |  |  |  |  |  |  |  | Encouragement | + |
|  |  |  |  |  |  |  |  |  |  |  |  |  |  | Covert control | ++ |
|  |  |  |  |  |  |  |  |  |  |  |  |  |  | Pressure to eat | - |
|  |  |  |  |  |  |  |  |  |  |  |  |  |  | Restriction | ++ |
|  |  |  |  |  |  |  |  |  |  |  |  |  |  | Monitoring | - |
| **Zohouri, 2004** | United Kingdom | RCS; 20 y FU | | Analysis of variance | Children from seven schools in south Northumberland | | 829 | 11-12 y | | Water consumption in grams/day | | 6 day and 15 day dietary record | | Time (2000 compared with 1980) | ++ |
|  |  |  |  |  |  |  |  |  |  |  |  |  |  | Sex (female) | + |
| *++ or - - : statistically significant positive or negative association; + or - : statistically non- significant positive or negative association; /: no association or unclear direction of association. †Abbreviations; AA=African-American; BMI=body mass index; CS=cross-sectional; FJ=fruit juice; FU=follow-up; HI=Hispanic; L=longitudinal; MA=Mexican American; RCS=repeated cross-sectional; SD=soft drink; SES=socioeconomic status; y=year. | | | | | | | | | | | | | | | |

| **Table S2: Risk of bias of studies included in the review on factors associated with water consumption among children.** | | | | | | | | |
| --- | --- | --- | --- | --- | --- | --- | --- | --- |
| **Author** | 1.Bias due to confounding* | 2.Bias in selection of participants into the study* | 3.Bias in classification of exposures* | 4.Bias due to departures from intended exposures* | 5.Bias due to missing data* | 6.Bias in measurement of outcomes* | 7.Bias in selection of the reported result* | Overall risk of bias* |
| **Ayala** | Serious | Serious | Moderate | NA | Low | Serious | Moderate | Serious |
| **Barraj** | Moderate | Serious | Low | NA | Low | Moderate | Moderate | Serious |
| **Beltrán-Aguilar** | Moderate | Moderate | Low | NA | Moderate | Serious | Moderate | Serious |
| **Bleich** | Moderate | Low | Low | Low | No info | Serious | Moderate | Serious |
| **Bougatsas** | Serious | No info | Low | NA | No info | Moderate | Moderate | Serious |
| **Campbell** | Moderate | Serious | Moderate | NA | No info | Serious | Moderate | Serious |
| **Campos** | Serious | Serious | Moderate | NA | No info | Serious | Moderate | Serious |
| **Cardon** | Moderate | Moderate | Low | NA | Low | Serious | Moderate | Serious |
| **Cockburn** | Serious | Moderate | Low | Low | Moderate | Serious | Moderate | Serious |
| **Condon** | Serious | Moderate | Moderate | NA | No info | Critical | Moderate | Critical |
| **Coppinger** | Moderate | Serious | Low | NA | No info | Moderate | Moderate | Serious |
| **Cullen** | Moderate | Serious | Moderate | NA | No info | Moderate | Serious | Serious |
| **Cunningham** | Moderate | Moderate | Moderate | NA | No info | Serious | Moderate | Serious |
| **Dai** | Moderate | Moderate | No info | NA | No info | Serious | Serious | Serious |
| **Danyliw** | Moderate | Moderate | Serious | NA | No info | Serious | Moderate | Serious |
| **De Craemer** | Moderate | Moderate | Low | NA | No info | Serious | Moderate | Serious |
| **Dodd** | Serious | Moderate | Low | NA | No info | Serious | Moderate | Serious |
| **Drewnowski** | Serious | Low | Low | NA | No info | Moderate | Moderate | Serious |
| **Dubuisson** | Moderate | Moderate | Moderate | NA | Moderate | Moderate | Moderate | Moderate |
| **Ebenegger** | Serious | Low | Low | NA | Moderate | Serious | Moderate | Serious |
| **Elmore** | Moderate | Moderate | Moderate | NA | Low | Serious | Serious | Serious |
| **Evans** | Moderate | Low | Moderate | NA | No info | Serious | Moderate | Serious |
| **Feferbaum** | Serious | Moderate | Low | NA | No info | Moderate | Moderate | Serious |
| **Fenandez-Alvira** | Serious | Moderate | Moderate | NA | No info | Moderate | Moderate | Serious |
| **Fernández-Alvira** | Moderate | Serious | Low | NA | No info | Serious | Moderate | Serious |
| **Franckle** | Moderate | Serious | Moderate | NA | No info | Serious | Moderate | Serious |
| **Guelinckx** | Moderate | Moderate | Low | NA | No info | Moderate | Moderate | Moderate |
| **Haroun** | Serious | Moderate | Low | Serious | No info | Low | Moderate | Serious |
| **Hoffman** | Serious | Serious | Moderate | NA | No info | Serious | Moderate | Serious |
| **Inhulsen** | Moderate | Moderate | Moderate | NA | Low | Serious | Moderate | Serious |
| **Jomaa** | Serious | Moderate | Low | NA | No info | Serious | Moderate | Serious |
| **Kaketiek** | Serious | Moderate | Serious | NA | Low | Moderate | Moderate | Serious |
| **Kant** | Moderate | Low | Serious | NA | No info | Serious | Serious | Serious |
| **Kaushik** | Serious | Serious | Moderate | NA | No info | Low | Moderate | Serious |
| **Lioret** | Moderate | Low | Low | NA | No info | Moderate | Moderate | Moderate |
| **Lora** | Serious | Serious | Moderate | NA | No info | Serious | Moderate | Serious |
| **Maffeis** | Serious | Serious | Low | NA | Low | Low | Moderate | Serious |
| **Makkes** | Serious | Moderate | Moderate | NA | No info | Serious | Moderate | Serious |
| **Mantziki** | Serious | Moderate | Serious | NA | Low | Serious | Serious | Serious |
| **Mantziki, 2015** | Serious | Moderate | Low | NA | No info | Serious | Moderate | Serious |
| **Milla Tobara** | Moderate | Serious | Moderate | NA | No info | Serious | Serious | Serious |
| **Murnan** | Moderate | Moderate | Moderate | NA | No info | Serious | Serious | Serious |
| **Papandreou** | Serious | Serious | Low | NA | No info | Moderate | Moderate | Serious |
| **Parsons** | Serious | Serious | Low | NA | No info | Serious | Moderate | Serious |
| **Patel, 2013** | Low | Moderate | Moderate | NA | No info | Moderate | Moderate | Moderate |
| **Patel, 2014** | Moderate | Moderate | Moderate | NA | Moderate | Serious | Moderate | Serious |
| **Petter** | Serious | Serious | Low | NA | No info | Serious | Moderate | Serious |
| **Piernas** | Serious | Low | Low | NA | No info | Moderate | Moderate | Serious |
| **Pinket, 2016a** | Moderate | Moderate | Moderate | NA | Low | Serious | Moderate | Serious |
| **Pinket, 2016b** | Moderate | Moderate | Moderate | NA | No info | Serious | Moderate | Serious |
| **Senterre** | Moderate | Moderate | Moderate | NA | Moderate | Moderate | Moderate | Moderate |
| **Sharma** | Moderate | Moderate | Moderate | NA | No info | Serious | Serious | Serious |
| **Sichert-Hellert** | Moderate | Moderate | Low | Low | No info | Low | Moderate | Moderate |
| **Sichieri** | Serious | Moderate | Serious | NA | No info | Serious | Serious | Serious |
| **Sleddens** | Moderate | Moderate | Moderate | Moderate | No info | Serious | Moderate | Serious |
| **Sohn** | Moderate | Moderate | Low | NA | Serious | Serious | Moderate | Serious |
| **Terry** | Serious | Moderate | Serious | NA | No info | Serious | Moderate | Serious |
| **Vereecken** | Moderate | Moderate | Low | NA | No info | Serious | Serious | Serious |
| **Vieux, 2016** | Moderate | Low | Moderate | NA | No info | Moderate | Moderate | Moderate |
| **Vieux, 2017** | Serious | Moderate | Moderate | NA | No info | Moderate | Moderate | Serious |
| **Wang** | Serious | Low | Low | NA | No info | Serious | Moderate | Serious |
| **Watowicz** | Serious | Low | Moderate | NA | Low | Serious | Moderate | Serious |
| **Zohouri** | Moderate | Moderate | Low | Moderate | Low | Moderate | Moderate | Moderate |
| *Risk of bias assessed by means of Risk Of Bias In Non-randomized Studies of Interventions (ROBINS-I) tool. Categories for each are low, moderate, serious and critical bias. | | | | | | | | |
